# Design and synthesis of purine nucleoside analogues for the formation of stable anti-parallel-type triplex DNA with duplex DNA bearing the ^5m^CG base pair†

**DOI:** 10.1039/d1ra02831f

**Published:** 2021-06-16

**Authors:** Ryotaro Notomi, Lei Wang, Shigeki Sasaki, Yosuke Taniguchi

**Affiliations:** Graduate School of Pharmaceutical Sciences, Kyushu University 3-1-1 Maidashi, Higashi-ku Fukuoka 812-8582 Japan taniguch@phar.kyushu-u.ac.jp; Graduate School of Pharmaceutical Sciences, Nagasaki International University 2825-7 Huis Ten Bosch Machi Sasebo Nagasaki 859-3298 Japan sigesasaki@niu.ac.jp

## Abstract

We herein demonstrated for the first time the direct recognition of duplex DNA bearing the 5-methyl-2′-deoxycytosine and 2′-deoxyguanosine base pair by triplex DNA formation. Triplex-forming oligonucleotides contained the novel artificial nucleoside analogues 2-amino-2′-deoxy-nebularine derivatives, and their molecular design, synthesis, and functional evaluation are described.

## Abstract 2

We herein demonstrated for the first time the direct recognition of duplex DNA bearing the 5-methyl-2′-deoxycytosine and 2′-deoxyguanosine base pair by triplex DNA formation.

## Graphical abstract

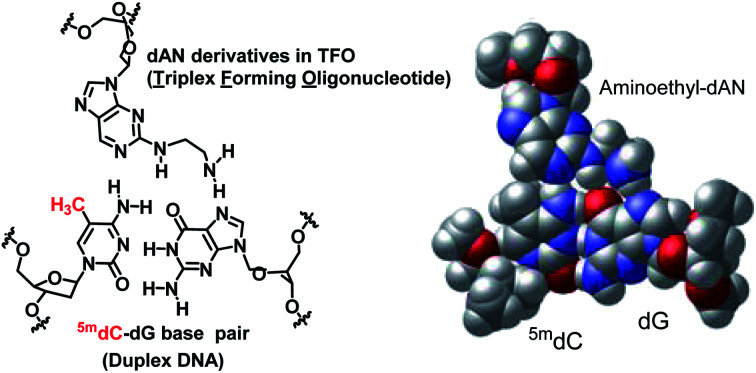

## 1. Introduction

**Fig. 1:**
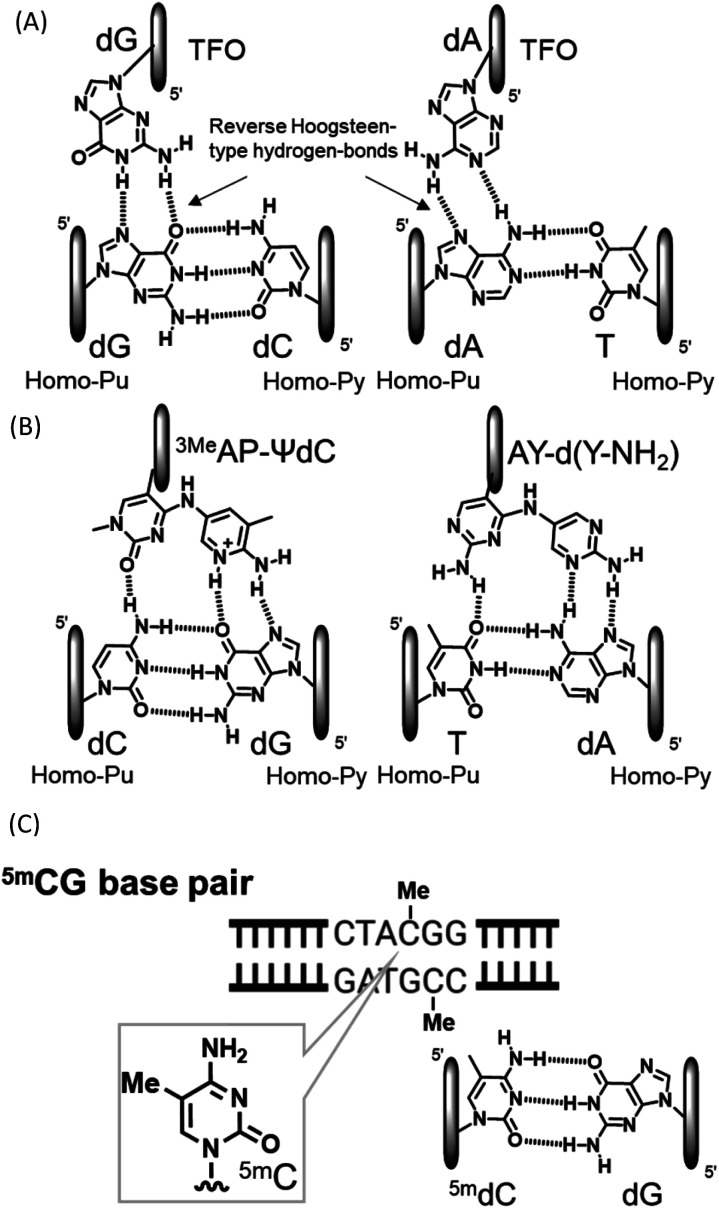
Triplet and duplex structures. (A) Natural-type base triplet. (B) Unnatural-type base triplet. (C) ^5m^CG base pair.

Compounds that directly interact with duplex DNA are of interest as gene-targeted tools. Among these compounds, the application of triplex-forming oligonucleotides (TFOs) has been the focus of research because they interact with each other from the major groove side of duplex DNA to form triplex DNA in a sequence-specific manner.^1–5^ Since the formation of triplex DNA enables the inactivation and activation of gene expression, recombination, and repair, this method may be a powerful tool for investigating genome-related events.^6–8^ However, the formation of stable triplex DNA with any sequence of duplex DNA is intrinsically limited. In purine-rich TFOs, guanine and adenine nucleobases in TFOs form two reverse Hoogsteen-type hydrogen bonds with guanine at the GC base pair and adenine at the AT base pair from the major groove side of the target duplex DNA, respectively (Fig. 1(A)). However, since the CG and TA base pairs have only one hydrogen bonding site at the major groove side of the cytosine and thymine nucleobases, it is not possible to form stable triplex DNA using TFOs comprising natural nucleosides. These inversion sites are called mismatch sites.^5^ We recently developed novel nucleoside analogues for the formation of stable triplex DNA with CG and TA mismatch sites and observed inhibitory effects on gene expression as an anti-gene effect (Fig. 1(B)).^7,9^ DNA methylation is an epigenetic process from the viewpoint of the control of gene expression.^10^ DNA methylation generally occurs at the 5 position of cytosine, resulting in 5-methylcytosine (^5m^C) (Fig. 1(C)).^11–14^ Since ^5m^C may form a base pair with guanine, similar to cytosine, it is a well-known gene expression control, mechanism that does not involve a change in the base sequence of a gene. These small chemical modifications have been confirmed in the CpG region and have been implicated in the pathogenesis of disease due to the abnormal methylation of the accumulated CpG sequence in the promoter region.^15^ A large number of detection and chemical reactants have been developed for the ^5m^C nucleobase in single-stranded DNA.^16–19^ Although sequence-specific ^5m^C recognizable molecules have not yet been developed in duplex DNA, they will contribute to detailed ^5m^C studies and related technologies. For example, it is expected to develop the DNA cleavage molecules, activation of transcription by recruitment of transcription factors based on the methylated cytosine, and demethylation inhibitors, and so on. The sequence-specific recognition of ^5m^C may be possible using triplex DNA formation: however, none of the artificial nucleoside analogues developed to date, including ours, recognize ^5m^CG base pairs.

**Fig. 2:**
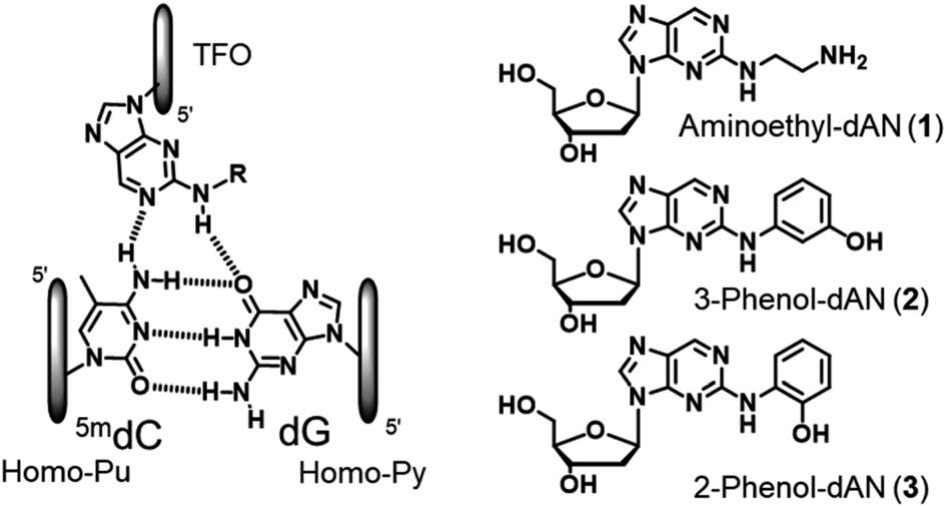
Design and structures of novel nucleoside analogues for the recognition of the ^5m^CG base pair.

Therefore, based on the artificial nucleoside analogues reported by our group, we designed novel artificial nucleoside analogues with a 2-amino-nebularine (dAN) skeleton. As a molecular design concept, we expected adjustments to the suitable position and stacking interactions with the neighboring nucleobases of the nebularine (dN) skeleton in TFOs,^20^ and introduced a hydrogen bonding unit into the amino group of dAN (Fig. 2). In the present study, we described the synthesis of the new dAN-based nucleoside analogues, aminoethyl-dAN (1), 3-phenol-dAN (2), and 2-phenol-dAN (3), for the recognition of the ^5m^CG base pair in duplex DNA. The triplex-forming abilities of TFOs with these nucleoside analogues were evaluated.

## 2. Results and discussion

### 2.1 Synthesis of dAN derivatives

**Scheme 1:**
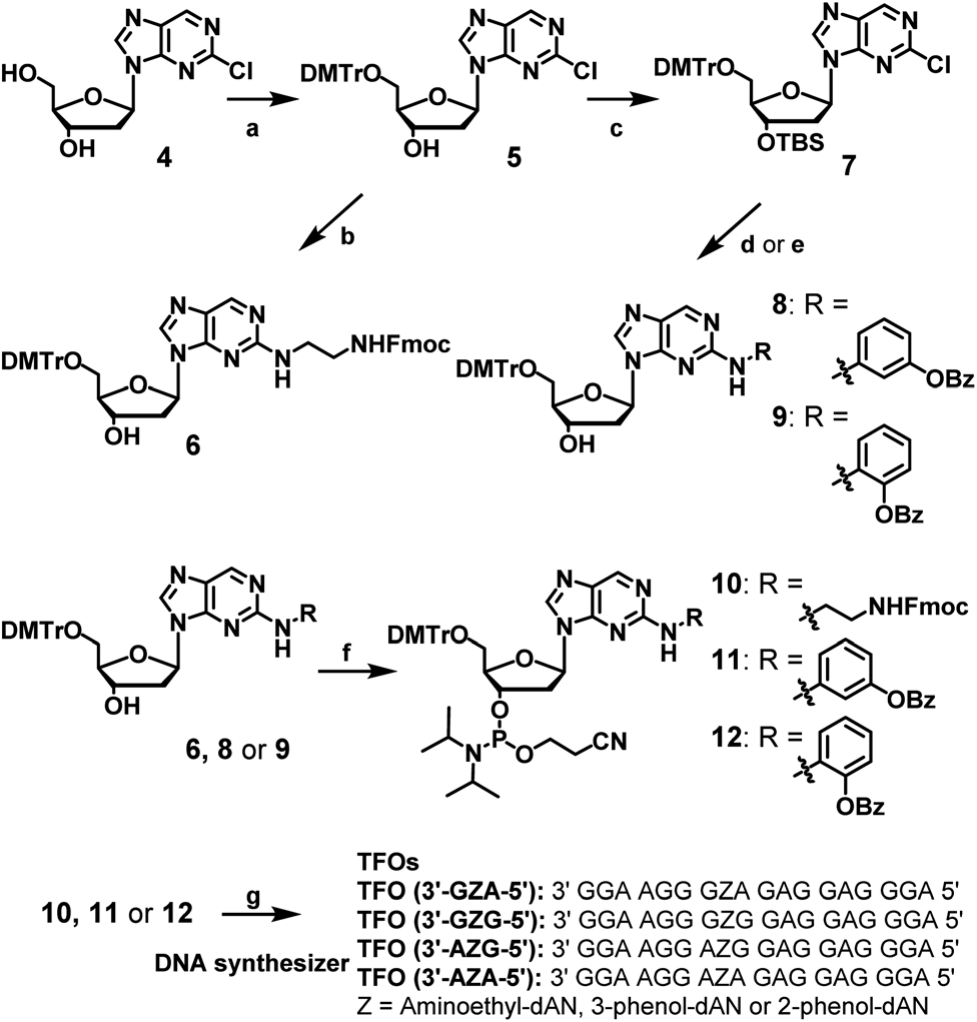
Synthesis of amidite compounds of new nucleoside analogues and TFOs. Reagents and conditions: (a) DMTrCl, pyridine, 92%, (b) (1) ethylenediamine, EtOH, 60 °C, (2) FmocCl, Et_3_N, CH_2_Cl_2_, 57% in 2 steps, (c) TBSCl, imidazole, CH_3_CN, 86%, (d) (1) PdAc_2_, xantphos, toluene, 3-aminophenyl benzoate, Cs_2_CO_3_, (2) Et_3_N–3HF, Et_3_N, THF, 80% in two steps, (e) (1) PdAc_2_, xantphos, toluene, 2-aminophenyl benzoate, NaO*t*Bu, (2) Bz_2_O, DMAP, (3) Et_3_N–3HF, Et_3_N, THF, 35% in three steps, (f) 2-cyanoethyl-*N*,*N*-diisopropylchlorophosphoramidite, DIPEA, CH_2_CH_2_, 0 °C, 49, 63, or 66% for 10, 11, or 12, respectively, (g) DNA automated synthesizer, followed by 28% ammonia solution at 55 °C for 12 h, HPLC purification, and then 5% acetic acid.

The synthesis of dAN derivatives is shown in Scheme 1. The 5′-hydroxyl group of 2-chloro-2′-deoxynebularine (4)^21^ was protected with a DMTr group (5). The aminoethyl unit was then introduced into the dN skeleton and the terminal amine was protected with the Fmoc group to give compound 6. On the other hand, the 3′-hydroxyl group was protected with a TBS group to give compound 7. 3-Amino-phenol derivatives were then coupled with 7, the TBS group of which was removed to give compound 8. Regarding 2-amino-phenol derivatives, a mixture of compounds with and without benzoyl groups was obtained in the coupling reaction. Therefore, the benzoyl-protecting reaction was performed using this mixture, and the TBS group was deprotected to give compound 9. Compounds (6, 8, or 9) were converted to the corresponding phosphoramidite units (10, 11, or 12, respectively).

### 2.2 Synthesis of TFOs with dAN derivatives

TFOs with dAN derivatives were synthesized using an automated DNA/RNA synthesizer under general reaction conditions, except for a coupling time of dAN derivatives of 10 minutes. After cleavage from CPG and the removal of the protecting groups using 28% ammonia solution, synthesized TFOs were purified by reverse-phase HPLC and identified by MALDI-TOF MS measurements (Table S1 and Fig. S3†).

### 2.3 Evaluation of triplex DNA formation with duplex DNA containing the ^5m^CG base pair

**Fig. 3:**
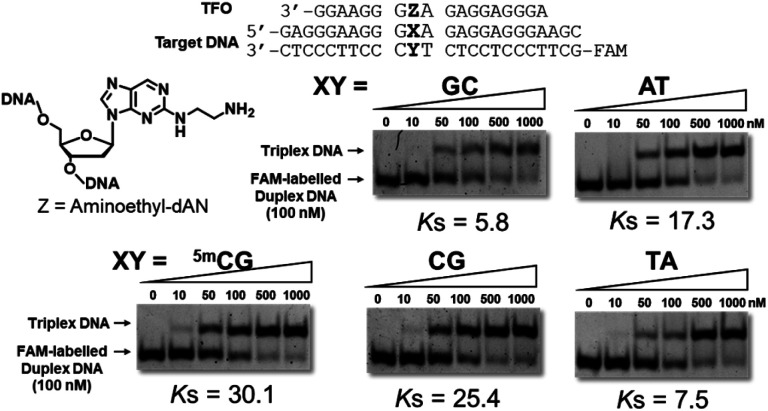
Gel results for the evaluation of triplex-forming abilities of synthesized TFOs. Triplex formation was performed in buffer containing 20 mM Tris–HCl and 20 mM MgCl_2_ at pH 7.5, FAM-labeled duplex DNA (24 bp; 100 nM) was incubated with increasing concentrations of TFO (18 mer; 0–1000 nM) at 37 °C, electrophoresis was performed using a 10% non-denatured polyacrylamide gel at 4 °C, *K*_s_ (10^6^ M^−1^) = [triplex]/([TFO][duplex]).

Triplex-forming abilities were assessed using a non-denatured polyacrylamide gel-shift assay with FAM-labeled duplex DNA, including the ^5m^CG, GC, AT, CG, or TA base pair, and synthesized TFOs. In brief, different concentrations of TFOs were added to a constant concentration of target duplex DNA in buffer containing 20 mM Tris–HCl and 20 mM MgCl_2_ at pH 7.5. After an incubation at 37 °C for 12 h, these samples were subjected to gel electrophoresis and detected by a fluorescence imager (Fig. 3 and S4†). Anti-parallel-type triplex DNA formation was observed as a slower-migrating band (Fig. S5†). Association constants (*K*_s_ values) were calculated from the fluorescence intensities of these bands. Mean *K*_s_ values are summarized in Table 1. This table also shows the association constants of previously reported artificial nucleoside analogues with the pyrimidine skeletons, ^3Me^AP-ΨdC and ^3Me^AP-d(Y-H), that recognize the CG base pair.

**Table 1:** Association constants (*K*_s_) for the formation of triplex DNA^a^

3′ NZN′ 5′	Z =	*K*_s_ (10^6^ M^−1^) for XY				
		^m^CG	GC	CG	AT	TA
3′ GZA 5′	Aminoethyl-dAN	30.1	5.8	25.4	17.3	7.5
	3-Phenol-dAN	0.3	0.5	5.3	17.4	4.1
	2-Phenol-dAN	5.6	5.4	10.3	9.1	9.1
	^3Me^AP-ΨdC	<0.1	1.8	32.6	<0.1	<0.1
	^3Me^AP-d(Y-H)	4.6	2.4	18.9	0.1	6.8
3′ GZG 5′	Aminoethyl-dAN	15.8	48.4	13.4	18.7	15.0
	3-Phenol-dAN	10.4	18.7	13.3	10.4	14.6
	2-Phenol-dAN	9.8	31.2	15.8	9.1	14.8
	^3Me^AP-ΨdC	<0.1	5.3	16.6	0.8	2.6
	^3Me^AP-d(Y-H)	7.5	19.2	39.4	12.6	24.2
3′ AZG 5′	Aminoethyl-dAN	3.8	3.3	3.3	3.0	7.4
	3-Phenol-dAN	26.7	10.1	18.0	19.4	43.3
	2-Phenol-dAN	11.6	6.9	20.8	10.8	14.0
	^3Me^AP-ΨdC	<0.1	1.8	19.4	<0.1	0.2
	^3Me^AP-d(Y-H)	5.1	0.4	8.4	0.2	5.1
3′ AZA 5′	Aminoethyl-dAN	<0.1	<0.1	0.1	<0.1	<0.1
	3-Phenol-dAN	0.2	<0.1	0.8	2.1	0.2
	2-Phenol-dAN	1.6	0.3	2.0	0.2	1.1
	^3Me^AP-ΨdC	<0.1	0.2	20.8	<0.1	<0.1
	^3Me^AP-d(Y-H)	<0.1	<0.1	1.8	<0.1	<0.1

a Conditions: FAM-labeled duplex DNA (24 bp; 100 nM) was incubated with increasing concentrations of TFO (18 mer; 0–1000 nM) in buffer containing 20 mM Tris–HCl and 20 mM MgCl_2_ at 37 °C and pH 7.5. Electrophoresis was performed using a 10% non-denatured polyacrylamide gel. *K*_s_ (106 M^−1^) = [triplex]/([TFO][duplex]). All values are the mean of three or more independent experimental values, errors are within 10%. Compounds of ^3Me^AP-ΨdC and ^3Me^AP-d(Y-H) from ref. 9*a* and 9*e* respectively.

In the sequences of 3′-dG and 5′-dA at the neighboring artificial nucleoside analogues, TFO with aminoethyl-dAN showed stable triplex formation with duplex DNA bearing the ^5m^CG base pair. TFOs with 3-phenol-dAN or 2-phenol-dAN did not stabilize triplex formation with duplex DNA bearing the ^5m^CG base pair. On the other hand, artificial nucleoside analogues with pyrimidine skeletons did not recognize the ^5m^CG base pair, suggesting that the purine skeleton contributed to increases in the stability of triplex DNA. Unfortunately, high selectivity was not obtained using this artificial nucleoside analogue. Nevertheless, we successfully recognized the ^5m^CG base pair within duplex DNA for the first time using stable triplex DNA formation. In the sequences of 3′-dG and 5′-dG, TFOs with dAN derivatives exerted moderate stabilizing effects for triplex formation with duplex DNA bearing the ^5m^CG base pair. These results on this sequence are very important for application and development as the ^5m^C-binding and/or -recognized molecule in CpG region. TFO with 3-phenyl-dAN showed stable triplex formation with duplex DNA bearing the ^5m^CG base pair. On the other hand, the formation of only a few triplex DNAs was observed in the dA sequences on both sides of phenol-dANs. When the 3′-side of aminoethyl-dAN was dA sequence, 3′-AZG-5′ and 3′-AZA-5′ sequences, the stability of TFOs for the formation of triplex DNA was low. These results revealed stacking interactions between two rings of the artificial nucleoside analogues during triplex DNA formation with adjacent bases in these sequences. However, consistent with previous findings,^9*c*,9*d*^ difficulties are associated with overcoming the issue of sequence dependence.

### 2.4 Optimized structure of the complex between dAN derivatives and the ^5m^CG base pair

**Fig. 4:**
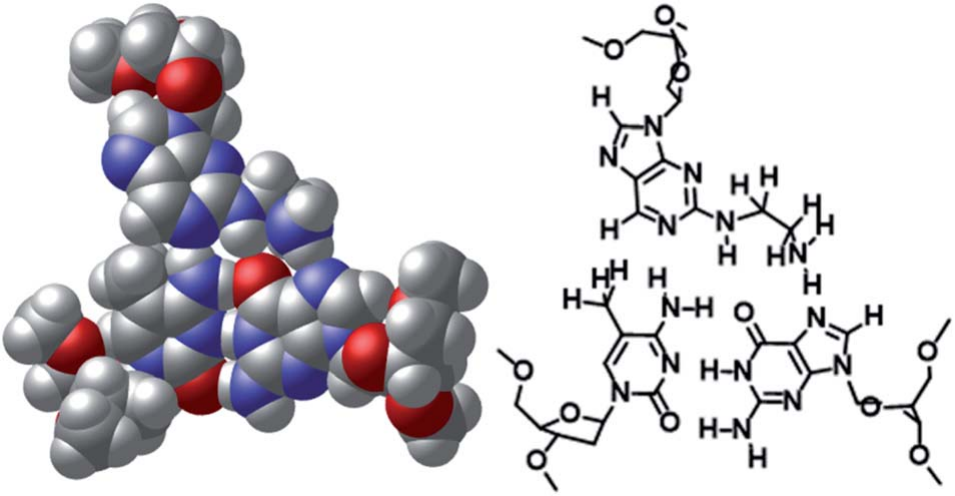
DFT at B3LYP/6-31 level-optimized structures of the aminoethyl-dAN/^5m^CG base triplet.

The non-natural-type triplet base pairs of aminoethyl-dAN/^5m^CG and phenol-dAN/^5m^CG were predicted using a computational study. The optimized base triplet of the CPK model and a traced picture are shown in Fig. 4 and S6.† According to these results, the amino-nebularine skeleton fits well at the junction of the ^5m^CG base pair and is located at a distance at which hydrogen bonding is possible, as designed for these molecules. The amino group of the aminoethyl unit is in a position at which it forms a hydrogen bond with nitrogen at the 7-position of the guanine base (Fig. 4). Similarly, the 3- or 2-phenol group is able to form a hydrogen bond with nitrogen at the 7-position or oxygen at the 6-position of the guanine base, respectively (Fig. S6†). Furthermore, the triplex DNA structure including aminoethyl-dAN/^5m^CG base triplet was predicted, and the picture focusing on three triplet base pair is shown in Fig. S7(A).† From either the 5′-side or the 3′-side of aminoethyl-dAN, it was observed that the dAN skeleton overlaps well with neighboring nucleobases within the TFO. No significant difference was observed between ^5m^CG and CG base pair as target base pair, but this will be important information for future molecular design (Fig. S7†).

## 3. Conclusions

We designed and synthesized the aminoethyl-dANs 3-phenol-dAN and 2-phenol-dAN, and evaluated the triplex-forming abilities of TFOs for target duplex DNA with the ^5m^CG base pair. By using the amino-nebraline (AN) skeleton as the basis of the artificial nucleoside analogue, it fit well into the junction of the ^5m^CG base pair. Furthermore, with the introduction of additional hydrogen bonding units, we succeeded in recognizing duplex DNA containing the ^5m^CG base pair by forming triplex DNA using TFOs with the sequences of 3′GZA5′, 3′GZG5′, and 3′AZG5′. We are now conducting a study to develop novel nucleoside analogues based on dAN derivatives for application to the recognition of ^5m^CG.

## 4. Experimental

### 4.1 Synthesis of dAN derivatives

^1^H-NMR (500 MHz), ^13^C-NMR (125 MHz), and ^31^P-NMR (202 MHz) spectra were generally recorded using a Bruker Ascend-500 spectrometer. High-resolution mass spectra were recorded by Bruker Daltonics micrOTOF II. MALDI-TOF/MS spectra were recorded by a Bruker Daltonics Microflex. UV-vis spectra were measured by a Beckman Coulter DU-800.

#### 4.1.1 Synthesis of 5′-*O*-(4,4′-dimethoxytrityl)-2′-deoxy-2-chloronebularine (5)

Under an argon atmosphere, pyridine (4.0 ml) was added to compound 4 (217 mg, 0.80 mmol). 4,4′-Dimethoxytrityl chloride (408 mg, 1.20 mmol) was added to this solution and stirred at room temperature for 2 h. Saturated NaHCO_3_ aqueous solution. (20 ml) was added to the reaction mixture and extracted with ethyl acetate (20 ml). The organic layer was washed with saturated NaCl solution (30 ml) and dried over Na_2_SO_4_. After filtration, the solvent was concentrated under a vacuum, and the crude mixture was purified by column chromatography (spherical silica gel 30 g, CH_2_Cl_2_/MeOH = 97/3 to 95/5) to give a yellow foam 5 (426 mg, 92% in 2 steps). ^1^H-NMR (500 MHz, CDCl_3_) *σ* (ppm) 8.84 (s, 1H), 8.14 (s, 1H), 7.65–7.71 (m, 1H), 7.36–7.40 (m, 2H), 7.24–7.30 (m, 6H), 6.76–6.81 (m, 4H), 6.44 (d, *J* = 6.50 Hz, 1H), 4.57–4.59 (m, 1H), 4.05–4.08 (m, 1H), 3.67 (s, 6H), 3.28–3.37 (m, 2H), 2.66–2.72 (m, 1H), 2.46–2.52 (m, 1H); ^13^C-NMR (125 MHz, CDCl_3_) *σ* (ppm) 158.79, 154.49, 152.64, 150.37, 149.91, 144.77, 144.60, 136.26, 135.70, 135.63, 133.96, 130.20, 128.23, 128.10, 127.20, 123.98, 113.40, 86.94, 86.68, 84.74, 72.49, 63.85, 55.41, 40.62, 31.77, 22.83, 14.30; ESI-HRMS (*m*/*z*): calcd for C_31_H_29_ClN_4_O_5_Na [M + Na]^+^: 595.1719, 597.1705, found: 595.1743, 597.1733.

#### 4.1.2 Synthesis of 5′-*O*-(4,4′-dimethoxytrityl)-2′-deoxy-2-*N*-[*N*-9-fluorenylmethyloxycarbonyl(aminoethyl)]nebularine (6)

Under an argon atmosphere, ethylenediamine (3.5 ml, 52.3 mmol) was added to compound 5 (300 mg, 0.523 mmol) in ethanol (5.2 ml) and heated under reflux at 60 °C for 13 h. The resulting reaction mixture was azeotroped with acetonitrile and toluene. Under an argon atmosphere, dichloromethane (5.2 ml) was added to the residue. 9-Fluorenylmethyl chloroformate (270 mg, 1.046 mmol) and triethylamine (220 μl, 1.569 mmol) were then added to this solution and stirred at room temperature for 1 h. The reaction mixture was concentrated under a vacuum. The residue was dissolved in ethyl acetate (20 ml), and the organic layer was washed with saturated NaHCO_3_ solution (20 ml) and saturated NaCl solution (20 ml). The organic layer was dried over Na_2_SO_4_, concentrated under a vacuum, and the residue was then purified by column chromatography (SHOKO 10 g, CH_2_Cl_2_/MeOH = 100/ to 96/4) to give a pale orange powder 6 (245 mg, 57% in 2 steps). ^1^H-NMR (500 MHz, CDCl_3_) *σ* (ppm) 8.65 (s, 1H), 7.81 (s, 1H), 7.69–7.77 (m, 4H), 7.58 (d, *J* = 7.44 Hz, 2H), 7.49–7.53 (m, 2H), 7.14–7.42 (m, 8H), 6.75–6.79 (m, 1H), 6.73 (d, *J* = 8.15 Hz, 4H), 6.26 (t, *J* = 6.32 Hz, 1H), 6.02 (bs, 1H), 4.74–4.79 (m, 1H), 4.41 (d, *J* = 6.84 Hz, 2H), 4.37 (d, *J* = 6.78 Hz, 2H), 4.14–4.22 (m, 2H), 4.06–4.09 (m, 1H), 3.74 (s, 6H), 3.46–3.54 (m, 1H), 3.28–3.43 (m, 4H), 2.84–2.90 (m, 1H), 2.39–2.45 (m, 1H); ^13^C-NMR (125 MHz, CDCl_3_) *σ* (ppm) 159.55, 158.63, 156.87, 144.68, 143.99, 143.94, 141.45, 141.08, 135.80, 130.15, 128.53, 128.23, 127.95, 127.85, 127.82, 127.19, 127.15, 127.00, 125.13, 125.07, 113.24, 86.50, 85.80, 84.00, 72.36, 66.91, 66.76, 63.91, 60.52, 55.32, 47.35, 47.31, 41.88, 41.29, 41.07, 40.42, 39.57, 31.70, 23.33, 22.76, 21.16, 14.31, 14.23; ESI-HRMS (*m*/*z*): calcd for C_48_H_46_N_6_O_7_Na [M + Na]^+^: 841.3320, found: 841.3356.

#### 4.1.3 Synthesis of 5′-*O*-(4,4′-dimethoxytrityl)-3′-*O*-(*tert*-butyldimethylsilyl)-2′-deoxy-2-chloronebularine (7)

Under an argon atmosphere, *tert*-butyldimethylsilyl chloride (689 mg, 4.57 mmol) and imidazole (415 mg, 6.09 mmol) were added to compound 5 (873 mg, 1.52 mmol) in acetonitrile (7.6 ml) and stirred at room temperature for 15 h. The reaction mixture was washed with saturated NaHCO_3_ solution (30 ml) and saturated NaCl solution (30 ml). The organic layer was dried over Na_2_SO_4_, concentrated under a vacuum, and the residue was then purified by column chromatography (spherical silica gel 10 g, hexane/EtOAc = 80/20 to 60/40) to give a white foam 7 (898 mg, 86%). ^1^H-NMR (500 MHz, CDCl_3_) *σ* (ppm) 8.94 (s, 1H), 8.27 (s, 1H), 7.35–7.38 (m, 2H), 7.26–7.28 (m, 4H), 7.17–7.24 (m, 3H), 6.76–6.80 (m, 4H), 6.43 (t, *J* = 6.41 Hz, 1H), 4.61–4.64 (m, 1H), 4.07–4.11 (m, 1H), 3.77 (s, 6H), 3.39 (dd, *J* = 4.17, 10.55 Hz, 1H), 3.32 (dd, *J* = 4.50, 10.56 Hz, 1H), 2.70–2.76 (m, 1H), 2.46 (ddd, *J* = 4.02, 6.30, 9.22 Hz, 1H), 0.86 (s, 9H), 0.05 (s, 3H), 0.02 (s, 3H); ^13^C-NMR (125 MHz, CDCl_3_) *σ* (ppm) 158.59, 154.33, 152.49, 150.23, 144.85, 144.46, 135.61, 135.57, 133.88, 130.04, 128.10, 127.87, 126.96, 113.18, 87.15, 86.62, 84.79, 77.24, 72.42, 63.22, 55.24, 40.77, 31.61, 25.77, 22.67, 18.00, 14.13, −4.65, −4.80; ESI-HRMS (*m*/*z*): calcd for C_37_H_43_ClN_4_O_5_SiNa [M + Na]^+^: 709.2583, 711.2559, found: 709.2580, 711.2580.

#### 4.1.4 Synthesis of 5′-*O*-(4,4′-dimethoxytrityl)-2′-deoxy-2-*N*-(3-phenylbenzoatyl)-nebularine (8)

Under an argon atmosphere, palladium(ii) acetate (15 mg, 0.067 mmol) and 4,5-bis(diphenylphosphino)-9,9-dimethylxanthene (78 mg, 0.134 mmol) were added to compound 7 (431 mg, 2.023 mmol) in toluene (3.4 ml) and freeze degassed. 3-Aminophenyl benzoate (463 mg, 0.674 mmol) and cesium carbonate (329 mg, 1.011 mmol) were added to this solution and stirred at 100 °C for 23 h. The reaction mixture was cooled at room temperature and filtered over Celite. The filtrate was concentrated under a vacuum, and the residue was then purified by column chromatography (Biotage 10 g, hexane/EtOAc = 50/50 to 30/70) to give a white foam. Under an argon atmosphere, triethylamine trihydrofluoride (320 μl, 1.962 mmol) and triethylamine (137 μl, 0.981 mmol) were added to this compound in tetrahydrofuran (4.9 ml) and stirred at room temperature for 15 h. Ethyl acetate (30 ml) was added to the reaction mixture and washed with saturated NaHCO_3_ solution (30 ml) and saturated NaCl solution (30 ml). The organic layer was dried over Na_2_SO_4_, concentrated under a vacuum, and the residue was purified by column chromatography (Biotage 10 g, hexane/EtOAc = 50/50 to 40/60) to give a brown powder 8 (344 mg, 80% in 2 steps). ^1^H-NMR (500 MHz, CDCl_3_) *σ* (ppm): 8.79 (s, 1H), 8.21 (d, *J* = 1.14 Hz, 1H), 8.19 (d, *J* = 1.34 Hz, 1H), 7.96 (s, 1H), 7.90 (t, *J* = 2.15 Hz, 1H), 7.62–7.66 (m, 1H), 7.49–7.54 (m, 2H), 7.33–7.37 (m, 4H), 7.16–7.25 (m, 8H), 6.88 (ddd, *J* = 0.84, 2.25, 7.21 Hz, 1H), 6.74–6.79 (m, 4H), 6.40 (t, *J* = 6.59 Hz, 1H), 4.55–4.59 (m, 1H), 4.09–4.15 (m, 1H), 3.76 (d, *J* = 1.21 Hz, 6H), 2.96–3.06 (m, 2H), 2.46–2.53 (m, 1H), 2.25–2.31 (m, 2H); ^13^C-NMR (125 MHz, CDCl_3_) *σ* (ppm): 158.57, 156.10, 151.79, 151.40, 149.84, 144.53, 140.87, 135.62, 130.19, 130.00, 129.58, 129.41, 128.71, 128.07, 127.8,9 126.96, 116.67, 113.33, 113.09, 86.54, 85.98, 55.76, 55.22, 40.34, 22.67, 14.14; ESI-HRMS (*m*/*z*): calcd for C_44_H_39_N_5_O_7_Na [M + Na]^+^: 772.2742, found: 772.2709.

#### 4.1.5 Synthesis of 5′-*O*-(4,4′-dimethoxytrityl)-2′-deoxy-2-*N*-(2-phenylbenzoatyl)-nebularine (9)

Under an argon atmosphere, palladium(ii) acetate (13 mg, 0.058 mmol) and 4,5-bis(diphenylphosphino)-9,9-dimethylxanthene (68 mg, 0.118 mmol) were added to compound 7 (431 mg, 2.023 mmol) in isopropanol (3.4 ml) and freeze degassed. 2-Aminophenyl benzoate (410 mg, 0.596 mmol) and sodium *tert*-butoxide (107 mg, 0.1113 mmol) were added to this solution and stirred at 80 °C for 42 h. The reaction mixture was cooled at room temperature and filtered over Celite. The filtrate was concentrated under a vacuum, and the residue was then purified by column chromatography (SHOKO 10 g, hexane/EtOAc = 70/30 to 50/50) to give a pale brown powder. Under an argon atmosphere, dichloromethane (2.5 ml), benzoic anhydride (55 mg, 0.246 mmol), and *N*,*N*-dimethylaminopyridine (3.0 mg, 0.024 mmol) were added to this compound and stirred at room temperature for 1 h. Ethyl acetate (20 ml) was added to the reaction mixture and washed with saturated NaHCO_3_ solution (20 ml) and saturated NaCl solution (20 ml). The organic layer was dried over Na_2_SO_4_, concentrated under a vacuum, and the residue was then purified by column chromatography (Yamazen 10 g, hexane/EtOAc = 70/30 to 50/50) to give a pale brown powder. Under an argon atmosphere, triethylamine trihydrofluoride (135 μl, 0.832 mmol) and triethylamine (58 μl, 0.416 mmol) were added to this compound in tetrahydrofuran (2.0 ml) and stirred at room temperature for 11 h. Ethyl acetate (20 ml) was added to the reaction mixture and washed with saturated NaHCO_3_ solution (20 ml) and saturated NaCl solution (20 ml). The organic layer was dried over Na_2_SO_4_, concentrated under a vacuum, and the residue was then purified by column chromatography (Kanto 60N, hexane/EtOAc = 50/50 to 30/70) to give a pale brown powder 9 (155 mg, 35%). ^1^H-NMR (500 MHz, CDCl_3_) *σ* (ppm) 8.73 (s, 1H), 8.36 (d, *J* = 7.12 Hz, 1H), 8.19 (dd, *J* = 1.30, 8.43 Hz, 2H), 7.92 (s, 1H), 7.63 (t, *J* = 7.71 Hz, 1H), 7.52 (t, *J* = 7.57 Hz, 2H), 7.37 (d, *J* = 7.21 Hz, 2H), 7.22–7.28 (m, 5H), 7.17–7.20 (m, 1H), 7.11 (dt, *J* = 1.52, 7.74 Hz, 1H), 6.78 (dd, *J* = 2.03, 8.90 Hz, 4H), 6.37 (t, *J* = 6.41 Hz, 1H), 5.29 (s, 1H), 4.59–4.63 (m, 1H), 4.07 (dd, *J* = 4.69, 8.98 Hz, 1H), 3.76 (s, 6H), 3.41 (dd, *J* = 4.76, 10.2 Hz, 1H), 3.31 (dd, *J* = 4.94, 10.2 Hz, 1H), 2.75–2.81 (m, 1H), 2.48–2.54 (m, 1H); ^13^C-NMR (125 MHz, CDCl_3_) *σ* (ppm) 164.71, 158.63, 156.29, 151.94, 149.83, 144.48, 141.25, 140.73, 135.60, 135.55, 133.98, 132.03, 130.33, 130.02, 129.36, 129.02, 128.78, 128.08, 127.96, 127.03, 126.33, 122.91, 122.48, 121.37, 113.25, 86.73, 85.69, 83.60, 72.65, 63.91, 55.25, 40.14, 31.62, 22.69, 14.15; ESI-HRMS (*m*/*z*): calcd for C_44_H_39_N_5_O_7_Na [M + Na]^+^: 772.2742, found: 772.2776.

#### 4.1.6 Synthesis of 5′-*O*-(4,4′-dimethoxytrityl)-3′-*O*-[2-cyanoethoxy(diisopropylamino)phosphino]-2′-deoxy-2-*N*-[*N*-9-fluorenylmethyloxycarbonyl(aminoethyl)]nebularine (10)

Under an argon atmosphere, *N*,*N*-diisopropylethylamine (204 μl, 1.172 mmol) and 2-cyanoethyl-*N*,*N*-diisopropylchlorophosphoramidite (130 μl, 0.586 mmol) were added to compound 6 (160 mg, 0.195 mmol) in dichloromethane (3.9 ml) at 0 °C for 4 h. Saturated NaHCO_3_ solution (20 ml) was added to the reaction mixture and then extracted with EtOAc (20 ml), washed with brine (20 ml), dried over Na_2_SO_4_, concentrated under a vacuum, and purified by column chromatography (Kanto 60N, CH_2_Cl_2_/MeOH = 50/1) to give a yellow foam. The foam was reprecipitated with cool hexane to give a yellow oil 10 (98 mg, 49%). ^1^H-NMR (500 MHz, CDCl_3_) *σ* (ppm) 8.65 (s, 1H), 7.85 (d, *J* = 3.65 Hz, 1H), 7.69–7.75 (m, 2H), 7.55 (d, *J* = 7.41 Hz, 2H), 7.32–7.40 (m, 5H), 7.23–7.29 (m, 5H), 7.18–7.22 (m, 3H), 6.72–6.76 (m, 4H), 6.24–6.31 (m, 1H), 4.72–4.80 (m, 1H), 4.40–4.45 (m, 2H), 4.22–4.29 (m, 1H), 4.17–4.21 (m, 1H), 3.75 (s, 6H), 3.55–3.65 (m, 3H), 3.46–3.54 (m, 2H), 3.36–3.43 (m, 2H), 3.24–3.35 (m, 4H), 2.73–2.77 (m, 1H), 2.56–2.63 (m, 2H), 2.43 (t, *J* = 6.50 Hz, 1H), 1.09–1.29 (m, 12H); ^13^C-NMR (125 MHz, CDCl_3_) *σ* (ppm) 158.55, 144.56, 144.00, 143.89, 141.35, 135.67, 130.16, 130.11, 128.28, 128.21, 127.85, 127.76, 127.71, 127.06, 126.92, 124.99, 124.95, 120.02, 119.97, 117.58, 116.92, 113.14, 86.34, 84.21, 77.28, 66.48, 63.59, 58.36, 58.30, 58.20, 58.16, 55.25, 47.30, 45.33, 43.34, 43.28, 41.84, 29.73, 24.65, 24.60, 24.52, 23.02, 23.01, 22.94, 22.92, 20.45, 20.39, 20.17, 20.11; ^31^P-NMR (202 MHz, CDCl_3_) *σ* (ppm) 148.64; ESI-HRMS (*m*/*z*): calcd for C_57_H_63_N_8_O_8_PNa [M + Na]^+^: 1041.4399, found: 1041.4398.

#### 4.1.7 Synthesis of 5′-*O*-(4,4′-dimethoxytrityl)-3′-*O*-[2-cyanoethoxy(diisopropylamino)phosphino]-2′-deoxy-2-*N*-(3-phenylbenzoatyl)-nebularine (11)

Under an argon atmosphere, *N*,*N*-diisopropylethylamine (139 μl, 0.800 mmol) and 2-cyanoethyl-*N*,*N*-diisopropylchlorophosphoramidite (89 μl, 0.400 mmol) were added to compound 8 (200 mg, 0.266 mmol) in dichloromethane (1.3 ml) at room temperature for 1.5 h. Saturated NaHCO_3_ solution (20 ml) was added to the reaction mixture and then extracted with EtOAc (20 ml), washed with brine (20 ml), dried over Na_2_SO_4_, concentrated under a vacuum, and purified by column chromatography (Biotage 10 g, hexane/EtOAc = 50/50 to 30/70) to give a pale yellow foam 11 (160 mg, 63%). ^1^H-NMR (500 MHz, CDCl_3_) *σ* (ppm) 8.78 (s, 1H), 8.21 (d, *J* = 7.24 Hz, 2H), 8.19 (d, *J* = 8.91 Hz, 1H), 7.61–7.66 (m, 1H), 7.46–7.59 (m, 4.5H), 7.36–7.41 (m, 2.5H), 7.31–7.36 (m, 1H), 7.27–7.30 (m, 2.5H), 7.15–7.25 (m, 3.5H), 6.86–6.90 (m, 1H), 6.73–6.79 (m, 4H), 6.39–6.43 (m, 1H), 4.67–4.77 (m, 1H), 4.25–4.31 (m, 1H), 3.73 (s, 6H), 3.54–3.72 (m, 4H), 3.30–3.43 (m, 2H), 2.89–2.97 (m, 1H), 2.65–2.71 (m, 0.5H), 2.54–2.62 (m, 1.5H), 2.41 (t, *J* = 6.53 Hz, 1H), 1.23–1.26 (m, 12H); ^13^C-NMR (125 MHz, CDCl_3_) *σ* (ppm) 171.19, 158.57, 156.04, 152.24, 151.43, 141.08, 135.66, 130.24, 130.10, 129.71, 129.50, 128.61, 128.20, 128.14, 127.86, 113.29, 113.06, 86.48, 60.42, 55.22, 21.05, 14.32, 14.10; ^31^P-NMR (202 MHz, CDCl_3_) *σ* (ppm) 149.02, 148.83; ESI-HRMS (*m*/*z*): calcd for C_53_H_56_N_7_O_8_PNa [M + Na]^+^: 972.3820, found: 972.3842.

#### 4.1.8 Synthesis of 5′-*O*-(4,4′-dimethoxytrityl)-3′-*O*-[2-cyanoethoxy(diisopropylamino)phosphino]-2′-deoxy-2-*N*-(2-phenylbenzoatyl)-nebularine (12)

Under an Ar atmosphere, *N*,*N*-diisopropylethylamine (139 μl, 0.800 mmol) and 2-cyanoethyl-*N*,*N*-diisopropylchlorophosphoramidite (89 μl, 0.400 mmol) were added to compound 9 (150 mg, 0.200 mmol) in dichloromethane (2.0 ml) at 0 °C for 1 h. Saturated NaHCO_3_ solution (20 ml) was added to the reaction mixture and then extracted with EtOAc (20 ml), washed with brine (20 ml), dried over Na_2_SO_4_, concentrated under a vacuum, and purified by column chromatography (Kanto 60N, hexane/EtOAc = 50/50 to 10/90) to give an orange foam 12 (126 mg, 66%). ^1^H-NMR (500 MHz, CDCl_3_) *σ* (ppm) 8.74 (d, *J* = 3.06 Hz, 1H), 8.39–8.45 (m, 1H), 8.19–8.22 (m, 1H), 7.99 (d, *J* = 6.85 Hz, 1H), 7.62–7.67 (m, 1H), 7.49–7.54 (m, 2H), 7.36–7.40 (m, 2H), 7.31 (d, *J* = 3.05 Hz, 1H), 7.22–7.29 (m, 8H), 7.18–7.22 (m, 1H), 7.08–7.13 (m, 1H), 6.75–6.80 (m, 4H), 6.40–6.45 (m, 1H), 4.64–4.71 (m, 1H), 4.26–4.32 (m, 1H), 3.77 (s, 3H), 3.75 (s, 3H), 3.56–3.66 (m, 2H), 3.31–3.40 (m, 2H), 2.57–2.71 (m, 2H), 2.56 (t, *J* = 6.28 Hz, 1H), 2.45 (t, *J* = 6.40 Hz, 1H), 1.10–1.27 (m, 12H); ^13^C-NMR (125 MHz, CDCl_3_) *σ* (ppm) 164.86, 158.77, 158.75, 156.37, 149.90, 144.65, 141.21, 140.70, 135.77, 135.72, 134.10, 132.27, 130.51, 130.29, 130.24, 129.48, 129.24, 128.93, 128.36, 128.3, 128.07, 127.13, 126.42, 122.85, 122.79, 122.65, 122.59, 121.27, 121.09, 113.37, 86.76, 83.99, 73.98, 63.85, 62.68, 60.59, 58.36, 55.42, 55.40, 43.56, 43.49, 43.46, 40.02, 24.82, 24.78, 24.73, 20.61, 20.44, 14.40; ^31^P-NMR (202 MHz, CDCl_3_) *σ* (ppm) 149.19, 148.78; ESI-HRMS (*m*/*z*): calcd. for C_53_H_56_N_7_O_8_PNa [M + Na]^+^: 972.3820, found: 972.3823.

### 4.2 Preparation of the TFOs

The 18-mer TFOs (3′-GGAAGGNZN’GAGGAGGGA-5′) (NN′ = GA, GG, AG and AA) incorporating dAN derivatives (Z = aminoethyl-dAN, 3-phenol-dAN and 2-phenol-dAN) were synthesized on a 1 μmol scale by an automated DNA synthesizer (Nihon Techno Service Co., Ltd.) using standard phosphoramidite chemistry. Cleavage from the resin was accomplished by an overnight treatment with 28% ammonium hydroxide at 55 °C for 5 h, followed by reversed-phase HPLC purification (column: Nacalai Tesque COSMOSIL 5C18-ARII, 10 × 250 mm, solvents: A: 0.1 M TEAA buffer, B: CH_3_CN, gradient: B for 10% to 40%/20 min, flow rate: 3.0 mL min^−1^, UV: 254 nm, column oven: 35 °C). The DMTr group was deprotected in 5% aqueous acetic acid at room temperature for 30 min, followed by reversed-phase HPLC purification (HPLC conditions: column: Nacalai Tesque COSMOSIL 5C18-ARII, 4.6 × 250 mm, solvents: A: 0.1 M TEAA buffer, B: CH_3_CN, B: 5% to 30%/20 min, flow rate: 1.0 mL min^−1^, UV: 254 nm, column oven: 35 °C). The structural integrity of the synthesized oligonucleotides was analyzed by MALDI-TOF MS (Table S1 and see an ESI†).

### 4.3 Evaluation of triplex formation using TFOs

FAM-labeled duplex DNA (24 bp, 100 nM) was incubated with increasing concentrations of TFOs (0–1000 nM) in buffer containing 20 mM Tris–HCl and 20 mM MgCl_2_ at 37 °C for 12 h at pH 7.5. Electrophoresis was performed at 4 °C using a 10% non-denatured polyacrylamide gel. The gel was visualized using the Luminoimage analyzer LAS-4000 (FUJIFILM), and the fluorescence intensity of each band was quantified to calculate association constants: *K*_s_ (10^6^ M^−1^) = [Triplex]/([TFO][Duplex]). Each *K*_s_ value was calculated from three independent experiments.^9*e*^

### 4.4 Computational calculation of base triplet structures

Base triplet structures with geometric optimization were created using the B3LYP density function with the 6-31G basis for the complex between the artificial nucleoside and ^5m^CG base pair *in vacuo*, as implemented in the Gaussian16W program. These optimized complex structures were visualized by CYLview.

## Conflicts of interest

There are no conflicts to declare.

## Supplementary Material

RA-011-D1RA02831F-s001
