# Supplementary material for: Design and synthesis of purine nucleoside analogues for the formation of stable anti-parallel-type triplex DNA with duplex DNA bearing the 5mCG base pair
Source: RSC Adv. 2021 Jun 16;11(35):21390–6. doi: 10.1039/d1ra02831f (PMC9034152; doi:10.1039/d1ra02831f)

Electronic Supplementary Material (ESI) for RSC Advances.
This journal is © The Royal Society of Chemistry 2021

Electronic supplementary information

Design and synthesis of purine nucleoside analogues for the formation of stable anti-parallel-type triplex DNA with duplex DNA bearing the ^{5m}CG base pair

Ryotaro Notomi, Lei Wang, Shigeki Sasaki and Yosuke Taniguchi

Contents:

1. ^{1}H-NMR and ^{13}C-NMR spectra of new compounds **5-9** (Figure S1) p2
2. ^{1}H-NMR, ^{13}C-NMR and ^{31}P-NMR spectra of new compounds **10**, **11** and **12** (Figure S2) P7
3. MALDI-TOF MS data of synthesized TFOs (Table S1) p11
4. MALDI-TOF MS charts of synthesized TFOs having dAN derivatives (Figure S3) p12
5. Gel results of triplex formation of TFOs having dAN derivatives (Figure S4) p15
6. Gel results of confirmation of triplex formation (Figure S5) p18
7. Optimized structures of the dAN-derivatives/^{5m}CG base triplet (Figure S6) p18
8. Molecular modeling of triplex DNA formation (Figure S7) p19

Figure S1 ^{1}H-NMR and ^{13}C-NMR spectra of new compounds 5-9.

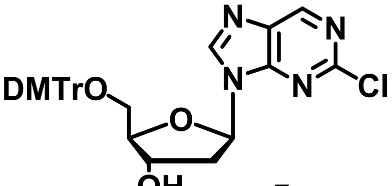

5

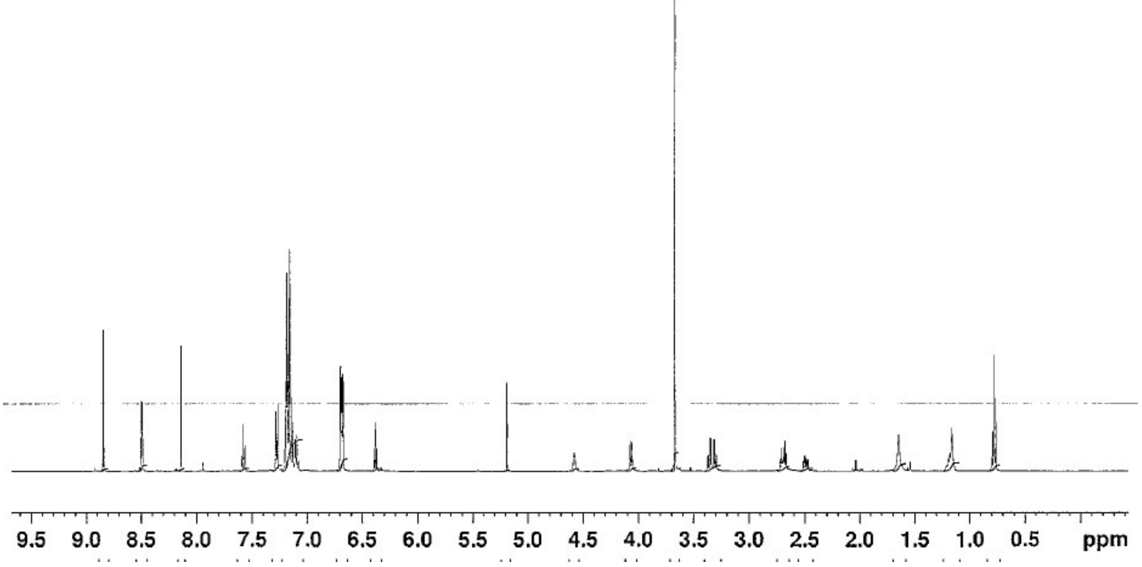

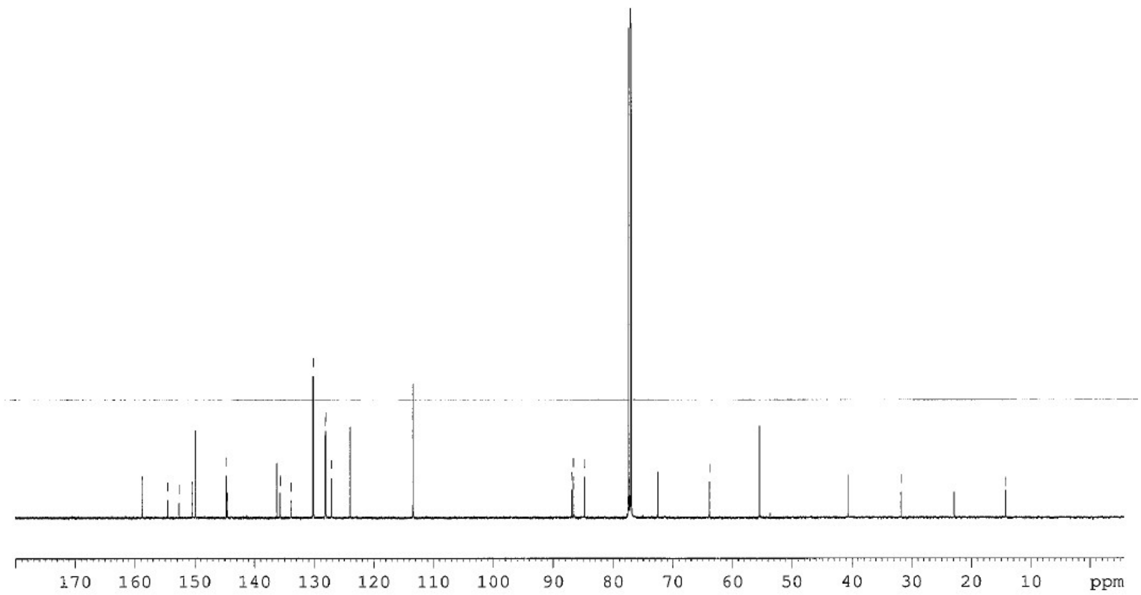

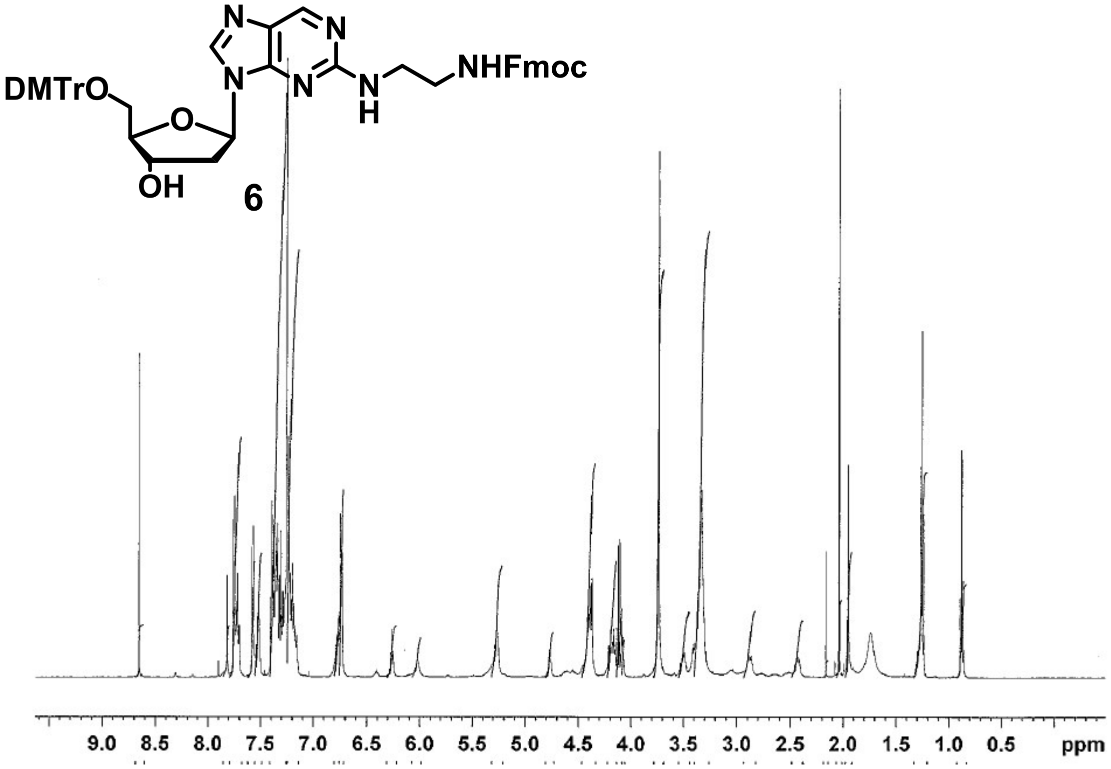
DMTrO
N
N
N
N
N
H
NHFmoc
O
OH
6
9.0 8.5 8.0 7.5 7.0 6.5 6.0 5.5 5.0 4.5 4.0 3.5 3.0 2.5 2.0 1.5 1.0 0.5 ppm

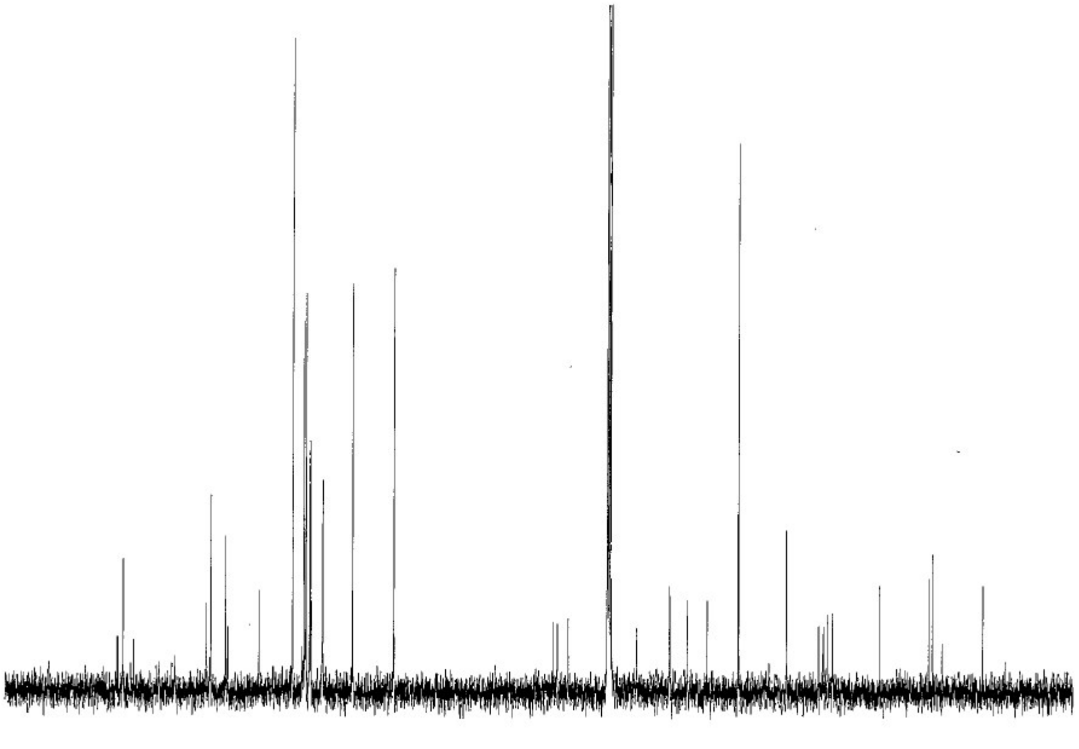
170 160 150 140 130 120 110 100 90 80 70 60 50 40 30 20 ppm

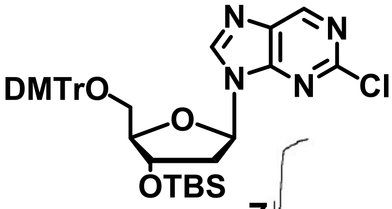
N
N
N
N
DMTrO
O
Cl
OTBS

7

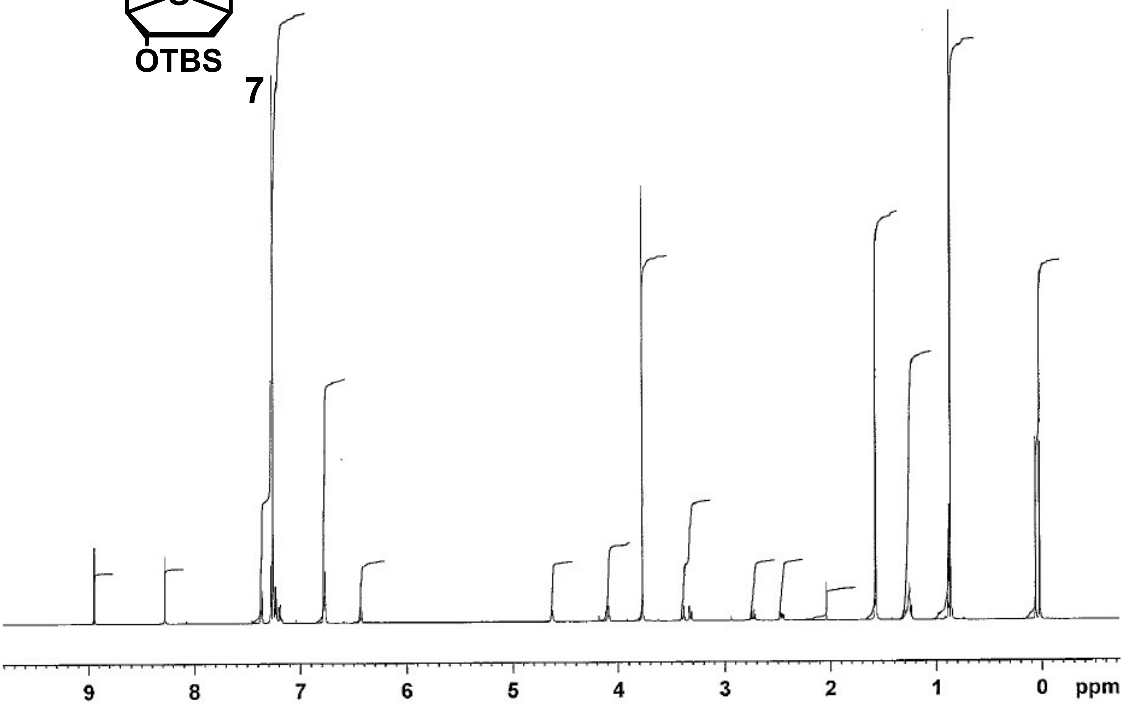
9 8 7 6 5 4 3 2 1 0 ppm

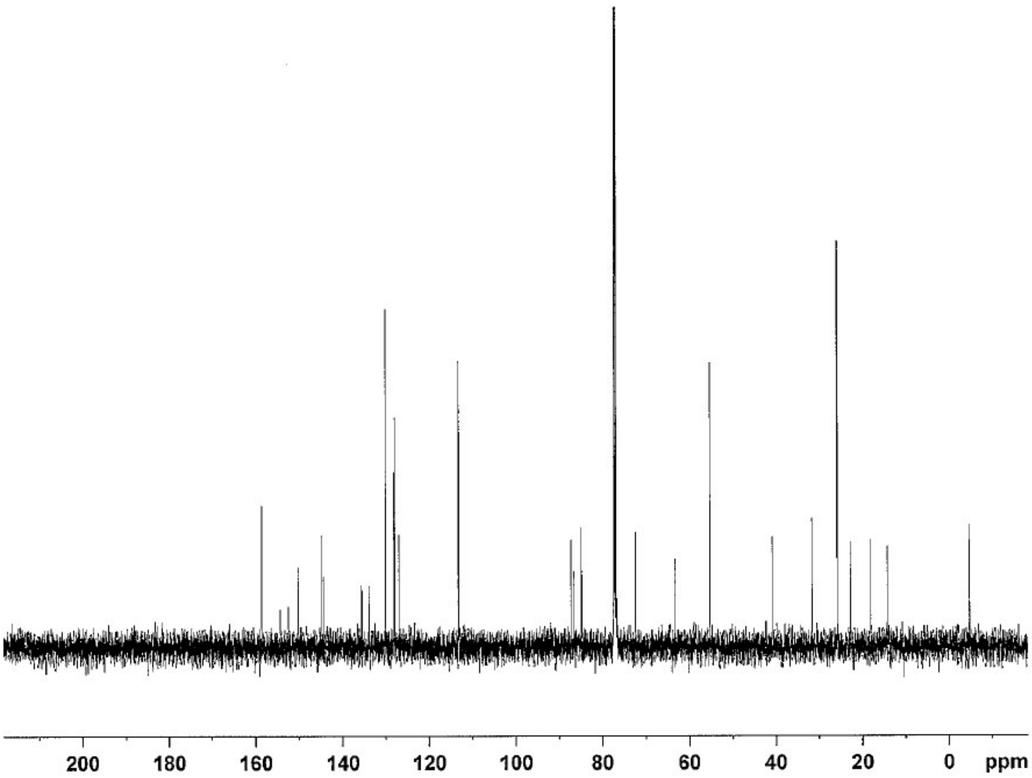
200 180 160 140 120 100 80 60 40 20 0 ppm

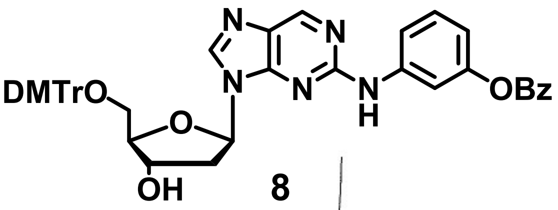

DMTrO
O
OH
N
N
N
N
N
H
OBz

8

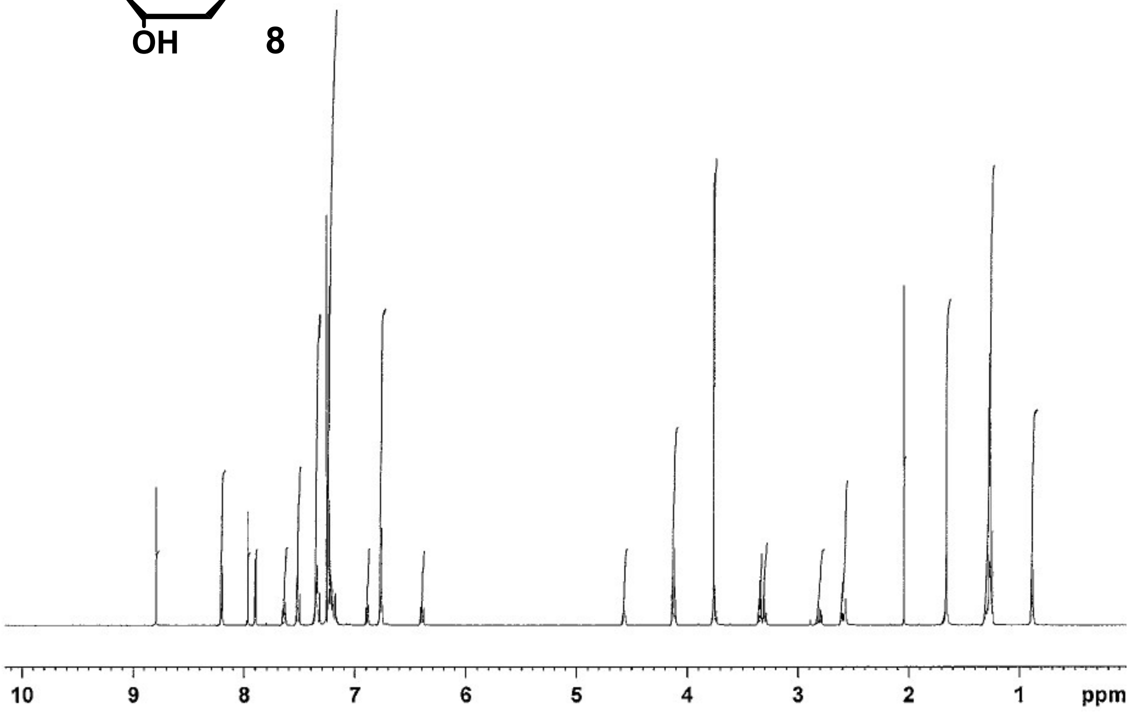

10 9 8 7 6 5 4 3 2 1 ppm

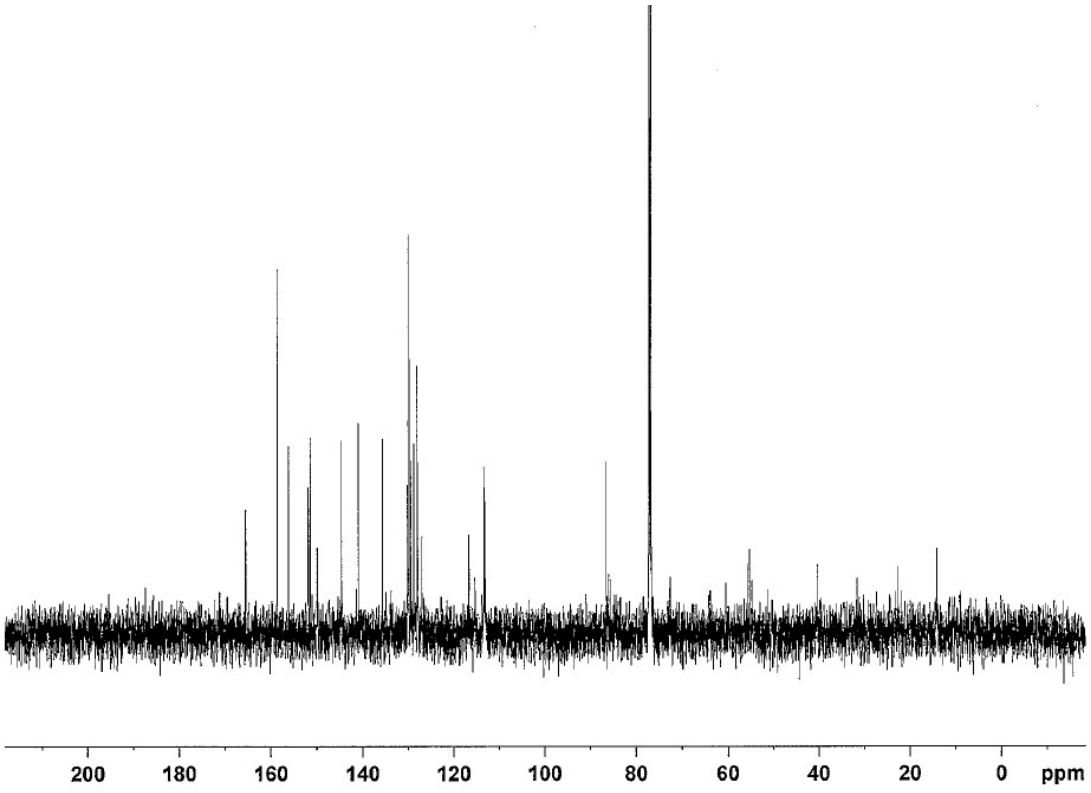

200 180 160 140 120 100 80 60 40 20 0 ppm

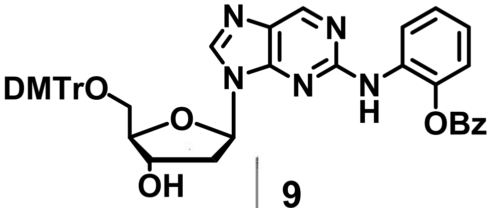
DMTrO
O
OH
N
N
N
N
N
H
OBz

9

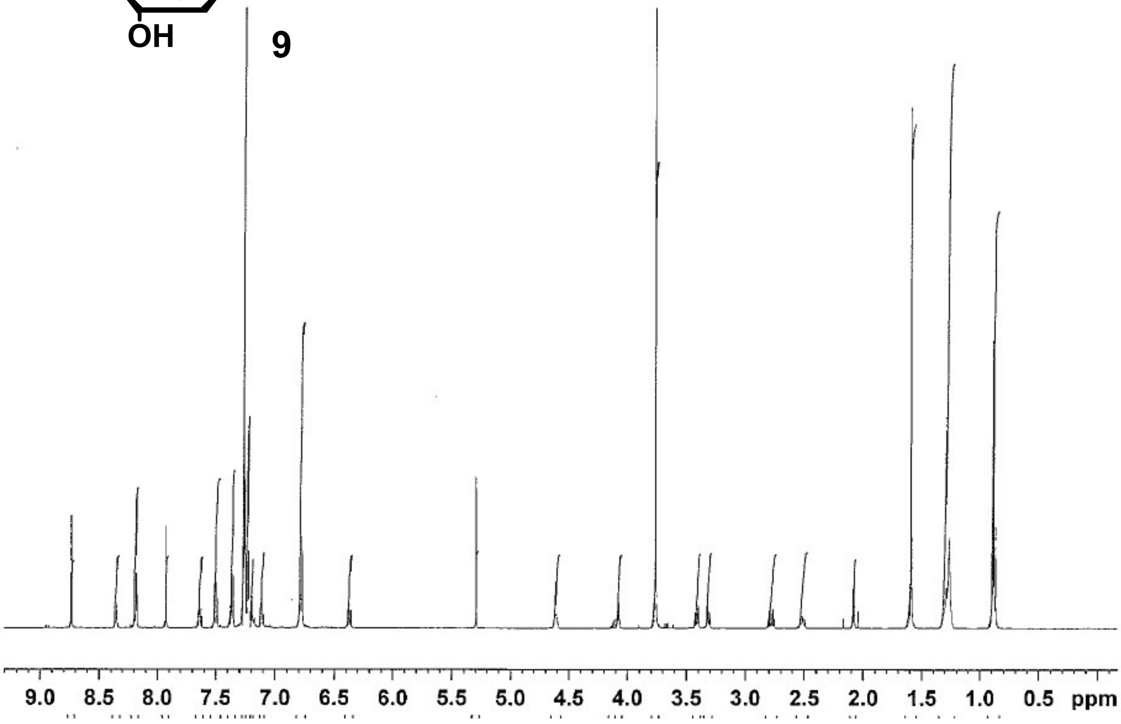
9.0 8.5 8.0 7.5 7.0 6.5 6.0 5.5 5.0 4.5 4.0 3.5 3.0 2.5 2.0 1.5 1.0 0.5 ppm

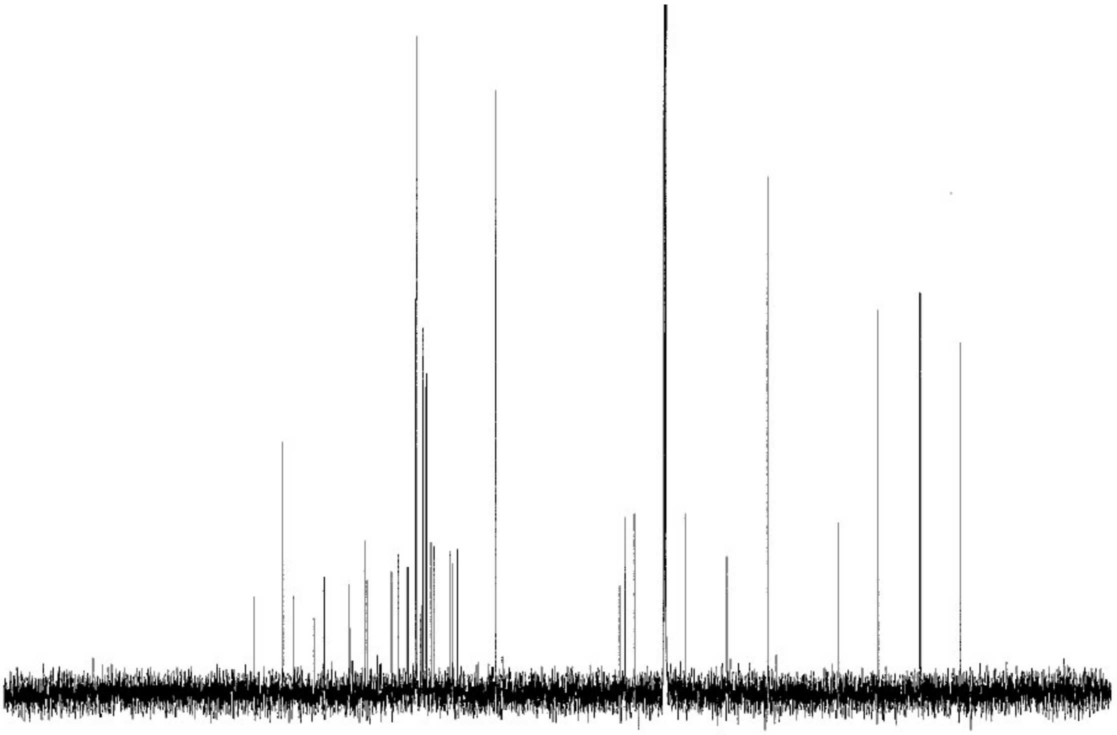
200 180 160 140 120 100 80 60 40 20 0 ppm

Figure S2 ^{1}H-NMR, ^{13}C-NMR and ^{31}P-NMR spectra of new compounds 10, 11 and 12.

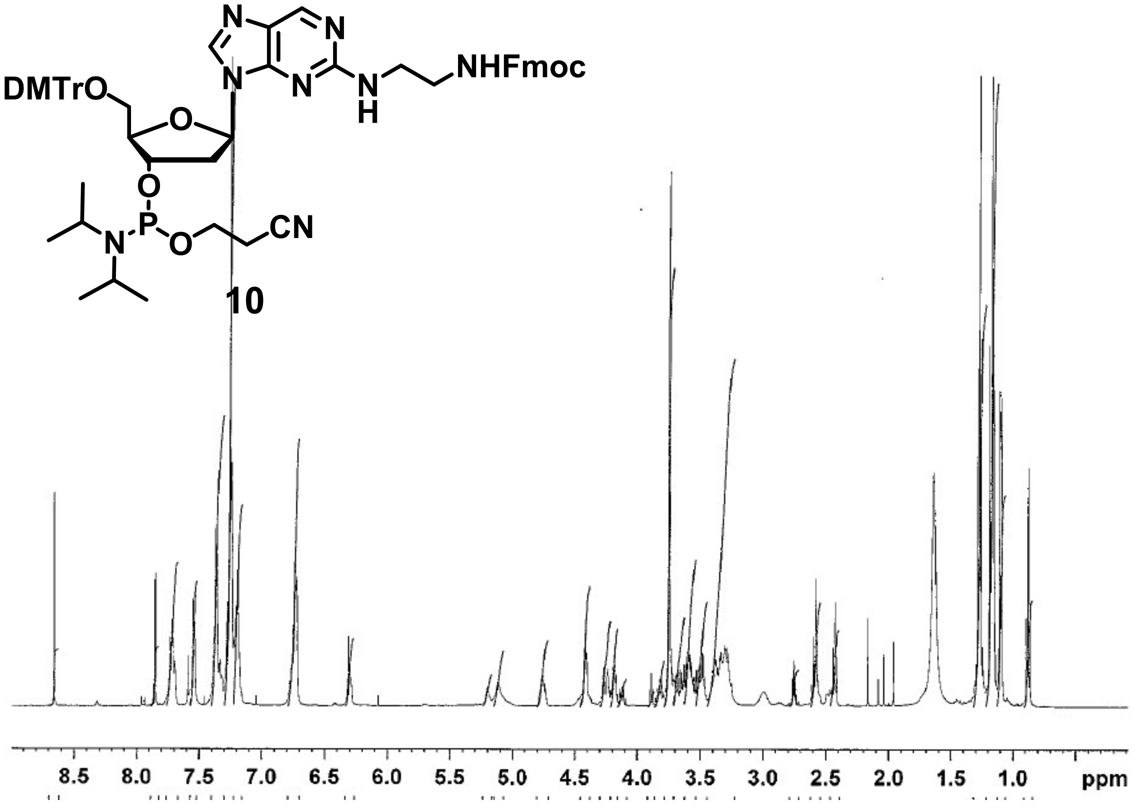

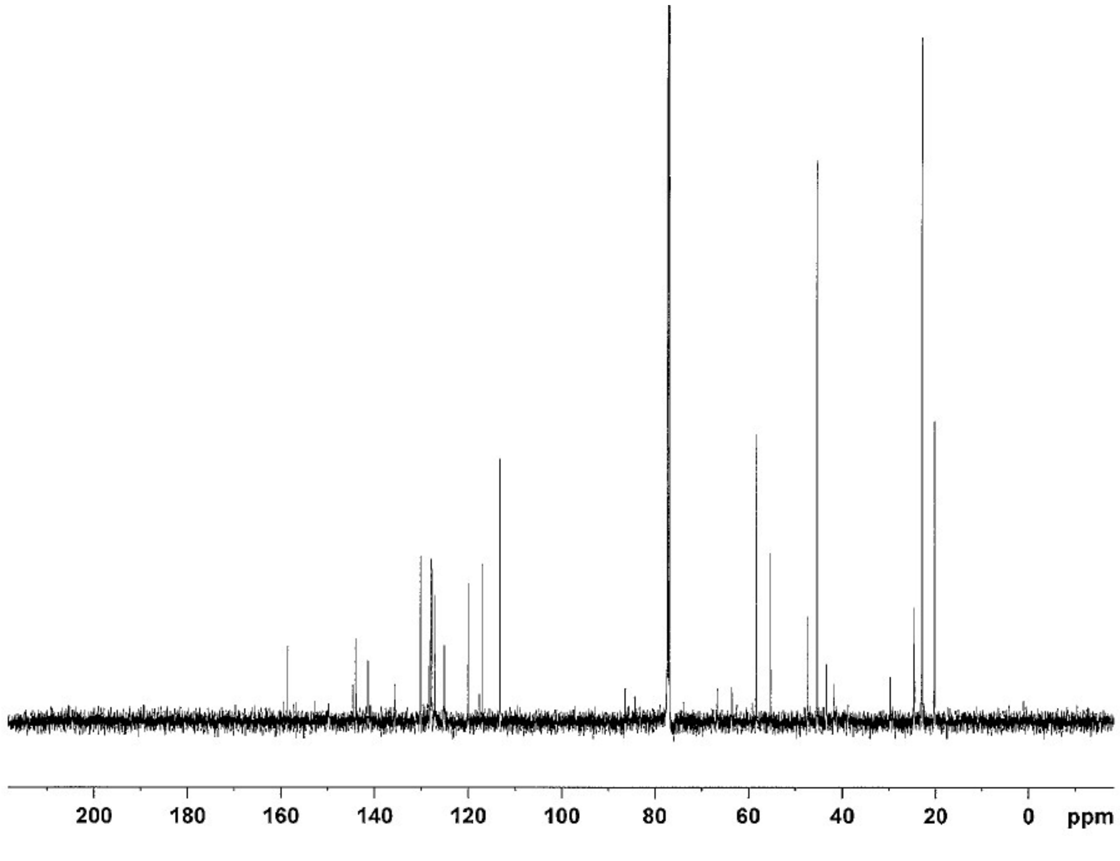

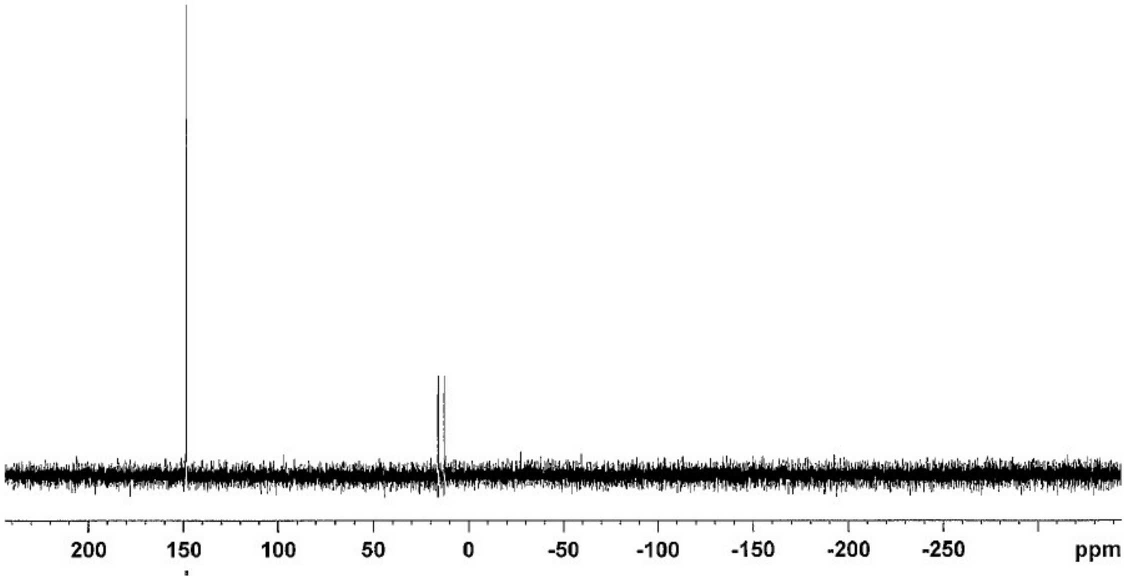
200 150 100 50 0 -50 -100 -150 -200 -250 ppm

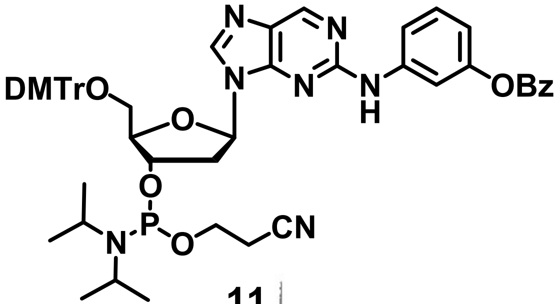
DMTrO
N
N
N
N
N
H
OBz
O
O
P
N
O
CN

11

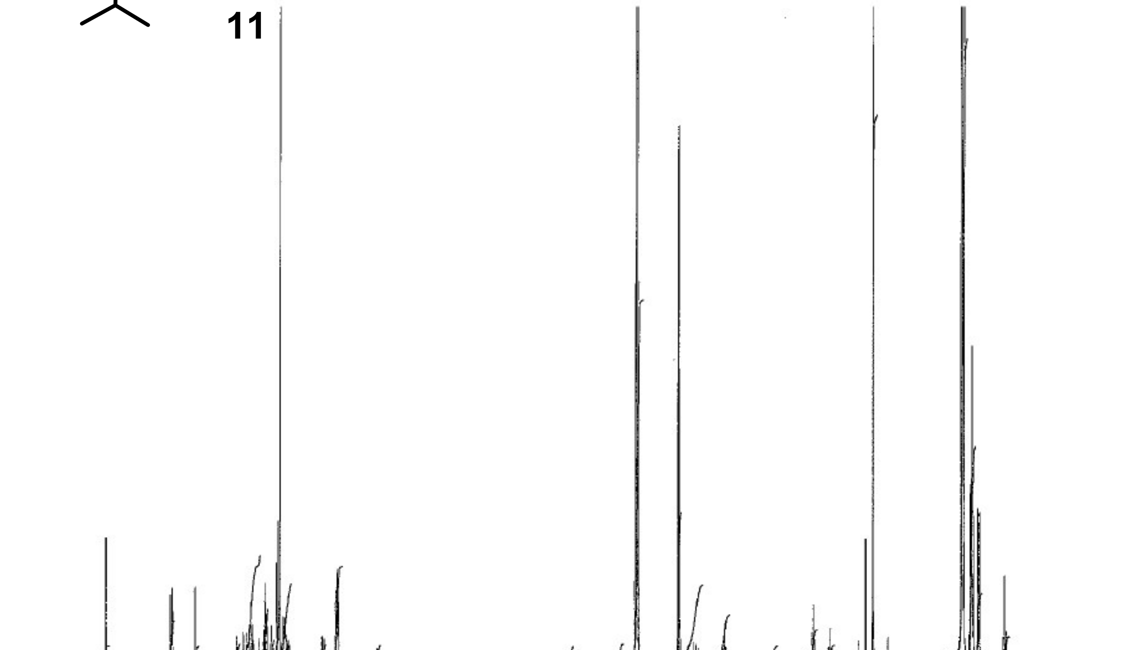
9.5 9.0 8.5 8.0 7.5 7.0 6.5 6.0 5.5 5.0 4.5 4.0 3.5 3.0 2.5 2.0 1.5 1.0 0.5 ppm

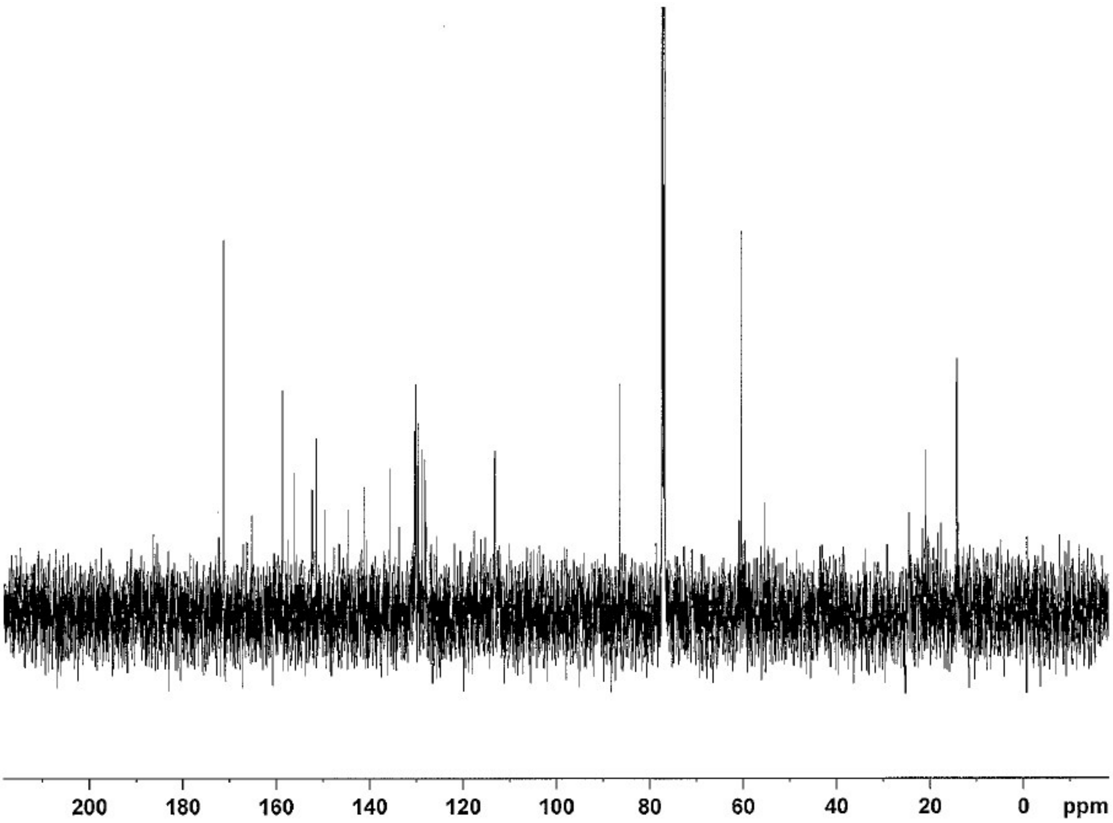
200 180 160 140 120 100 80 60 40 20 0 ppm

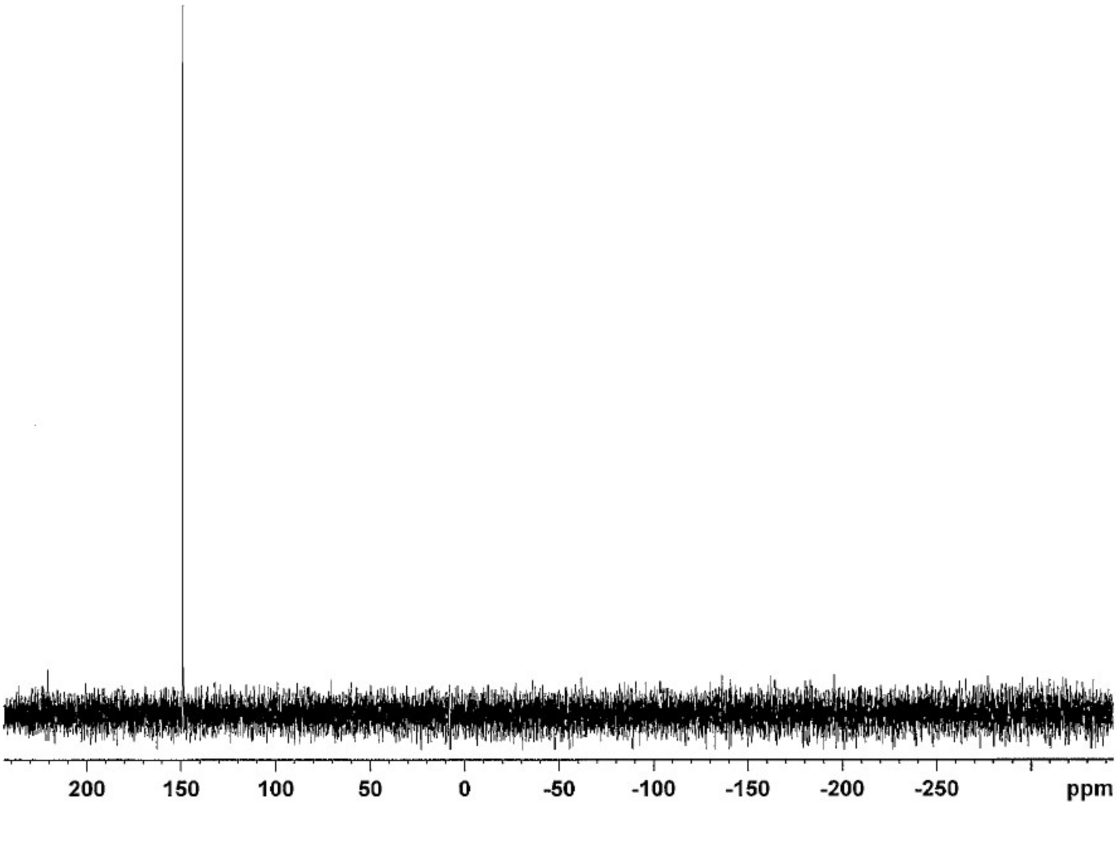
200 150 100 50 0 -50 -100 -150 -200 -250 ppm

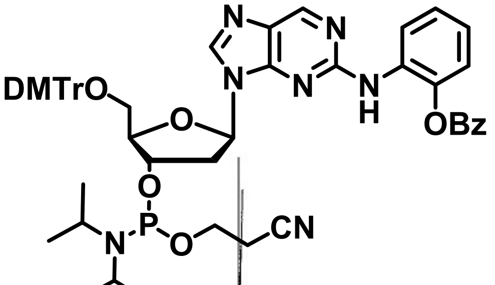
DMTrO
N
N
N
N
N
H
OBz
O
O
P
N
O
CN

12

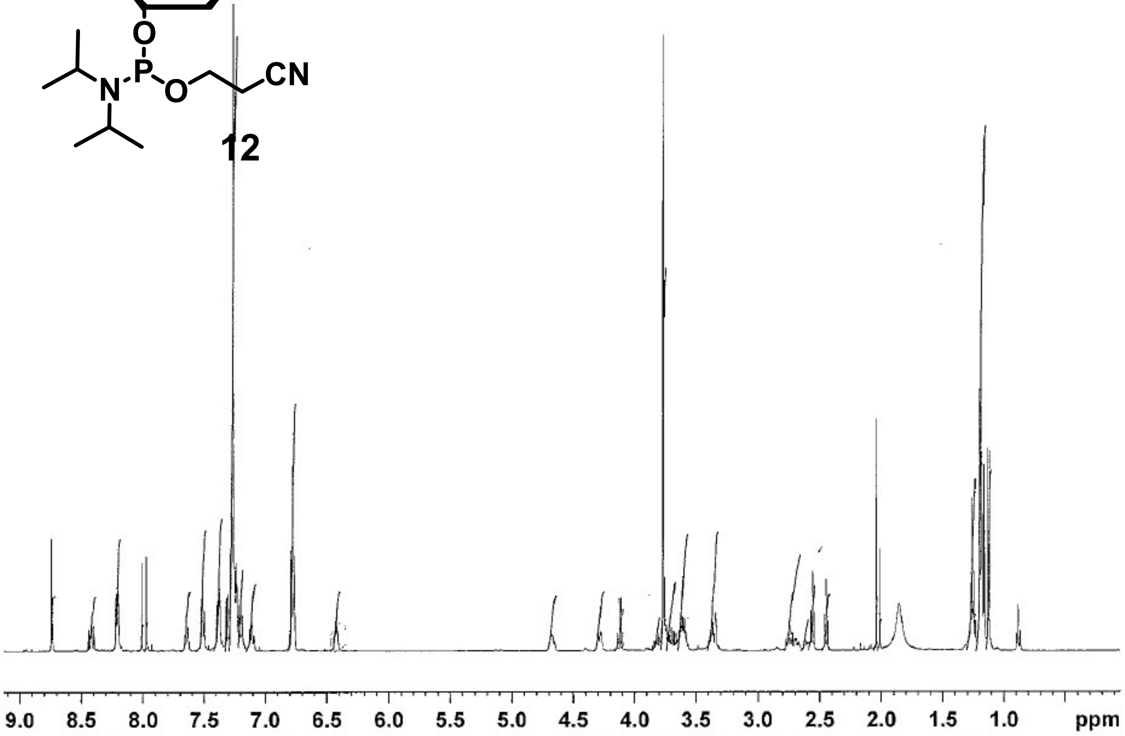
9.0 8.5 8.0 7.5 7.0 6.5 6.0 5.5 5.0 4.5 4.0 3.5 3.0 2.5 2.0 1.5 1.0 ppm

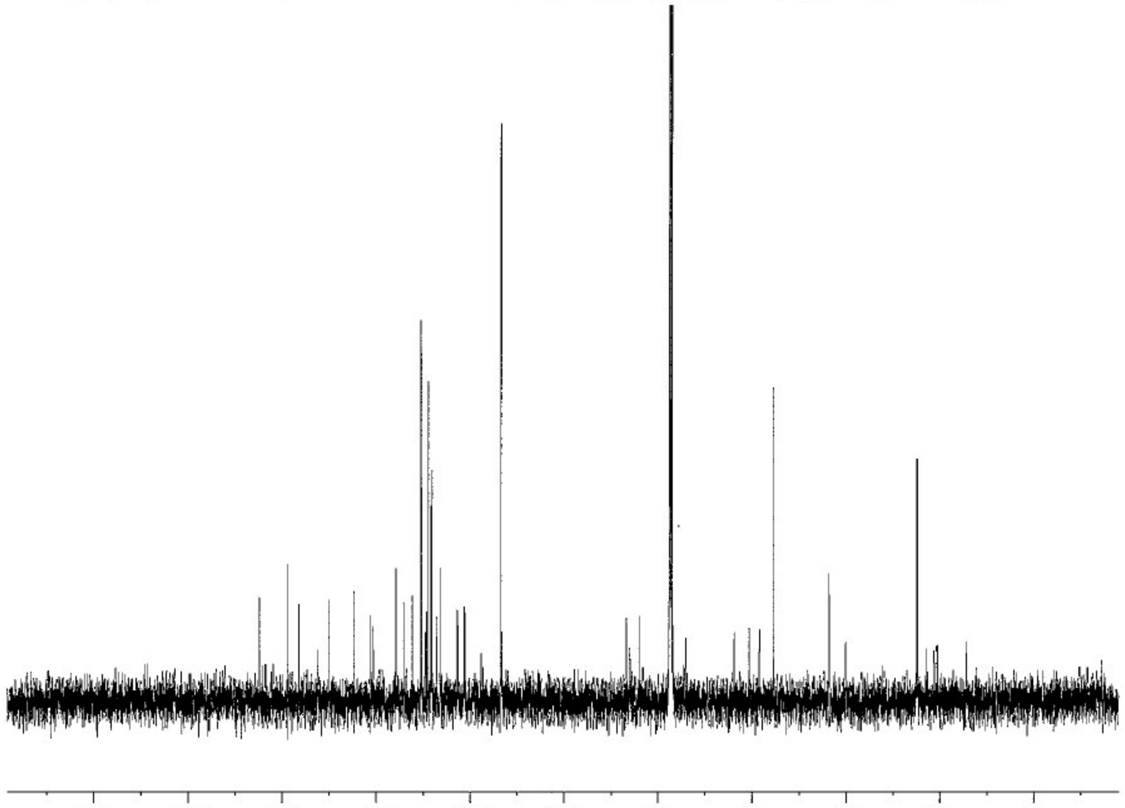
200 180 160 140 120 100 80 60 40 20 0 ppm

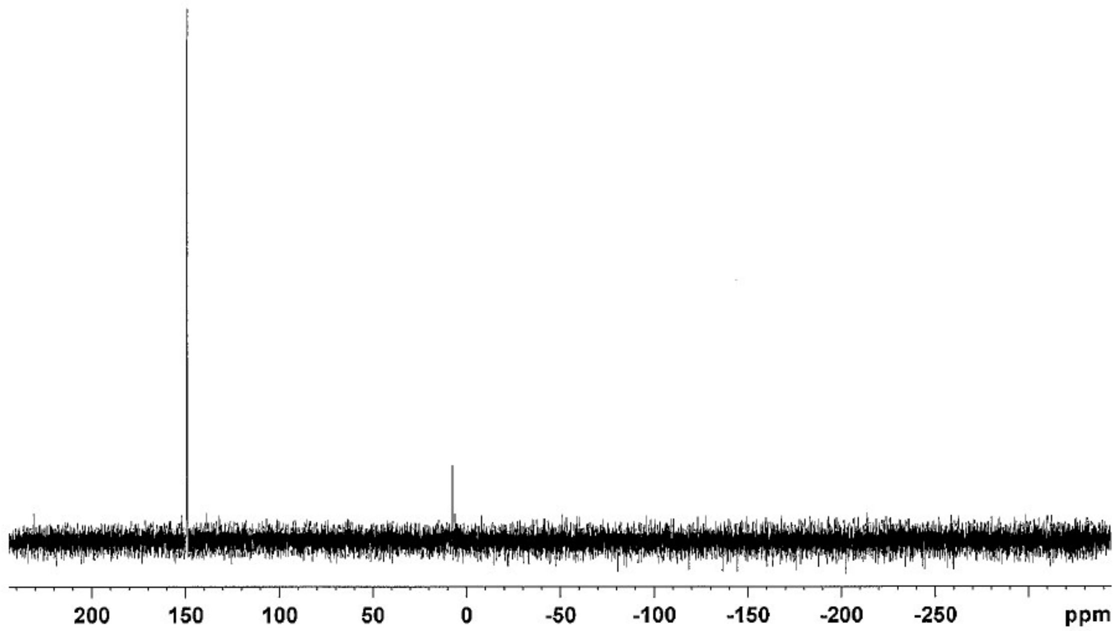

Table S1. MALDI-TOF MS results for synthesized TFOs.[a]

dAN derivatives	sequences	calcd. [M-H]$^-$	found
Aminoethyl-dAN	3'-GZA-5'	5791.06	5794.29
	3'-GZG-5'	5807.05	5809.70
	3'-AZG-5'	5791.06	5792.84
	3'-AZA-5'	5775.06	5777.47
3-Phenol-dAN	3'-GZA-5'	5840.04	5840.21
	3'-GZG-5'	5856.04	5855.39
	3'-AZG-5'	5840.04	5840.13
	3'-AZA-5'	5824.05	5822.79
2-Phenol-dAN	3'-GZA-5'	5840.04	5841.41
	3'-GZG-5'	5856.04	5856.98
	3'-AZG-5'	5840.04	5841.94
	3'-AZA-5'	5824.05	5824.54

[a] The structural integrity of all synthesized TFOs (3' GGA AGG *NZN'* GAG GAG GGA 5') was analyzed by MALDI-TOF MS (m/z). *NZN'* = GZA, GZG, AZG and AZA.

Figure S3 MALDI-TOF MS charts of synthesized TFOs having dAN derivatives.

3'-G Aminothyl-dAN A-5'

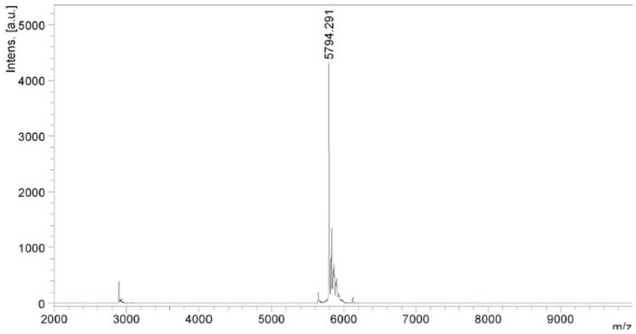

3'-G Aminothyl-dAN G-5'

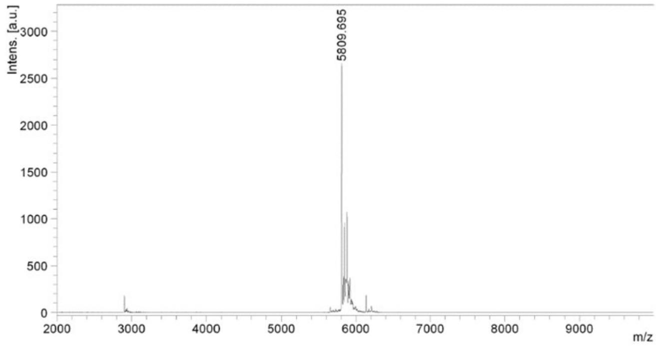

3'-A Aminothyl-dAN G-5'

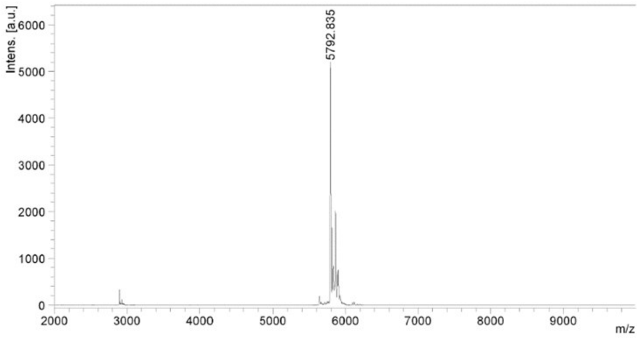

3'-A Aminothyl-dAN A-5'

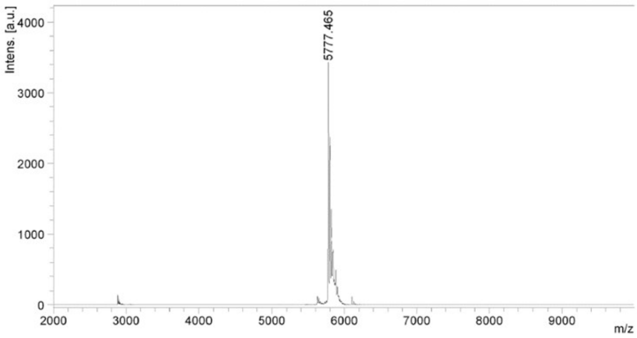

3’-G 3Phenol-dAN A-5’

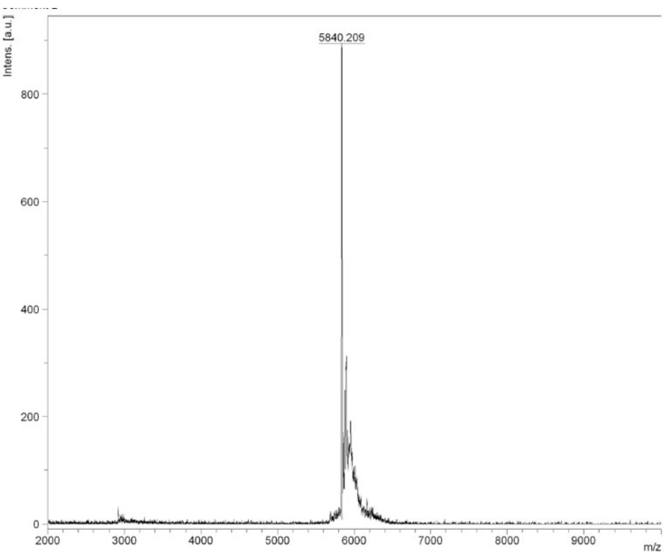

3’-G 3Phenol-dAN G-5’

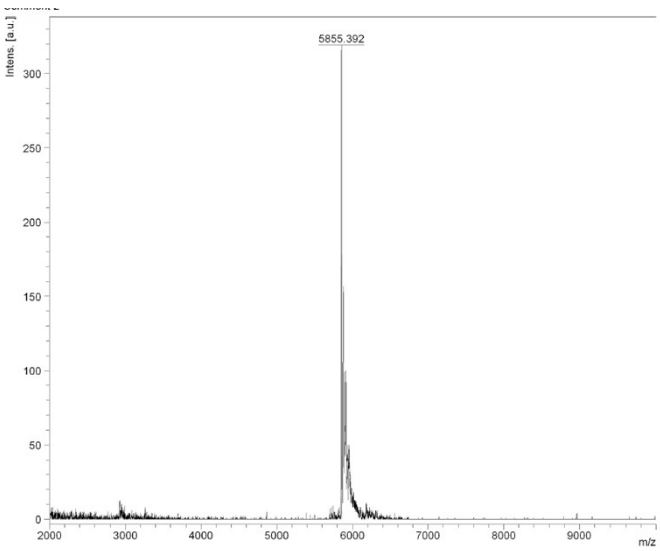

3’-A 3Phenol-dAN G-5’

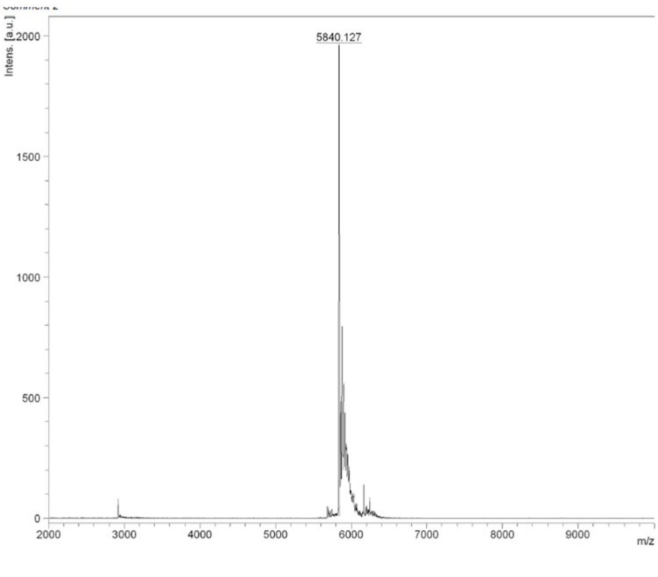

3’-A 3Phenol-dAN A-5’

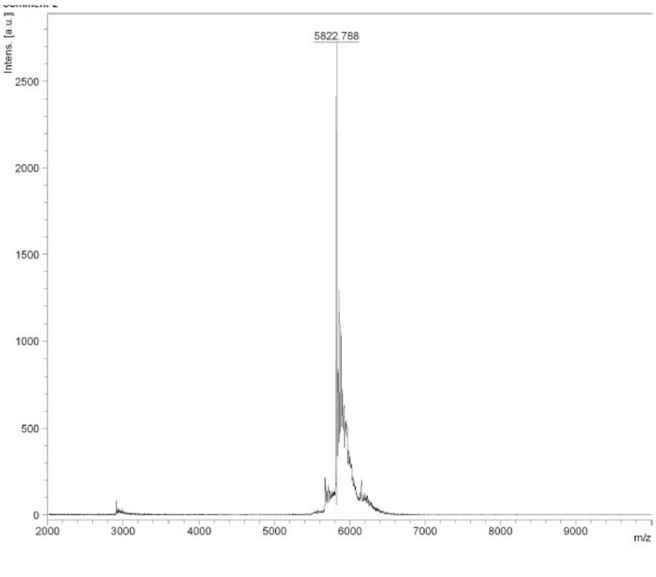

3’-G 2Phenol-dAN A-5’

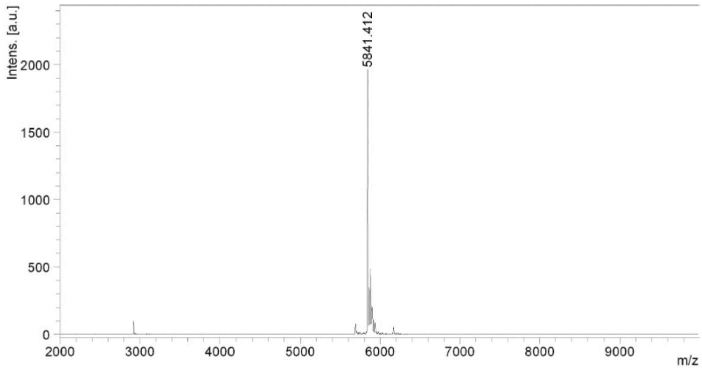

3’-G 2Phenol-dAN G-5’

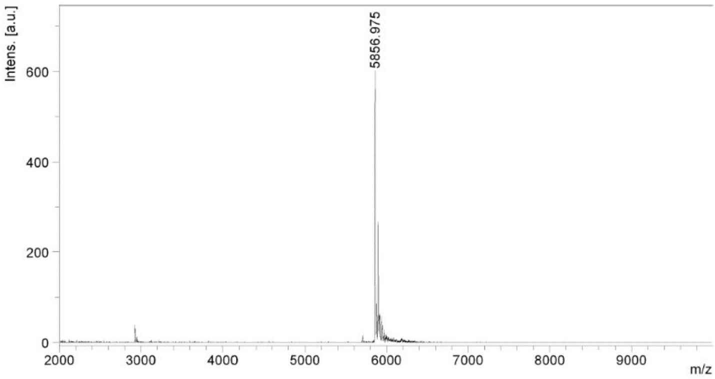

3’-A 2Phenol-dAN G-5’

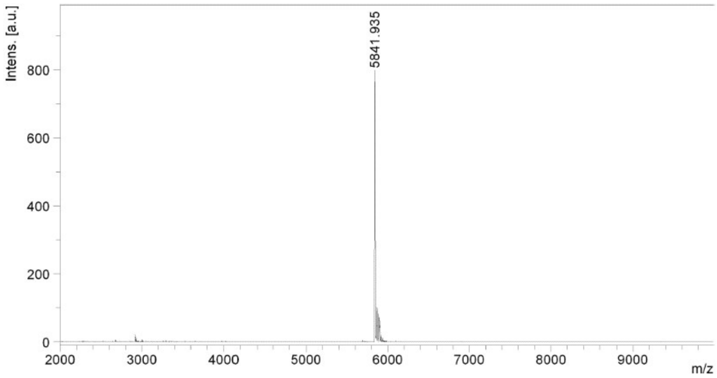

3’-A 2Phenol-dAN A-5’

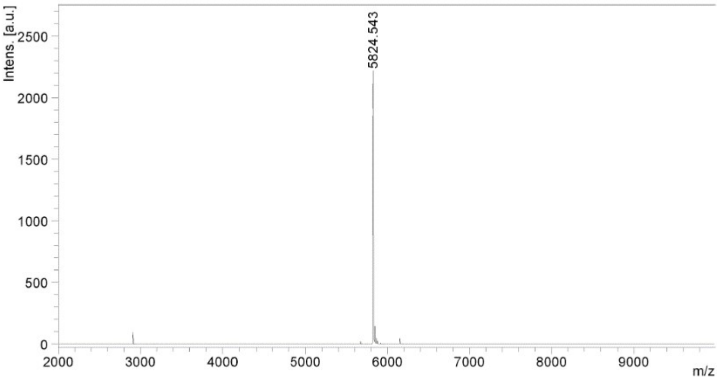

Figure S4 Gel results of triplex formation of TFOs having dAN derivatives.

TFO	3′-GGAAGG NZN′ GAGGAGGGA
Target DNA	5′-GAGGGAAGG NXN′ GAGGAGGGAAGC
	3′-CTCCCTTCC MYM′ CTCCTCCCTTCG-FAM

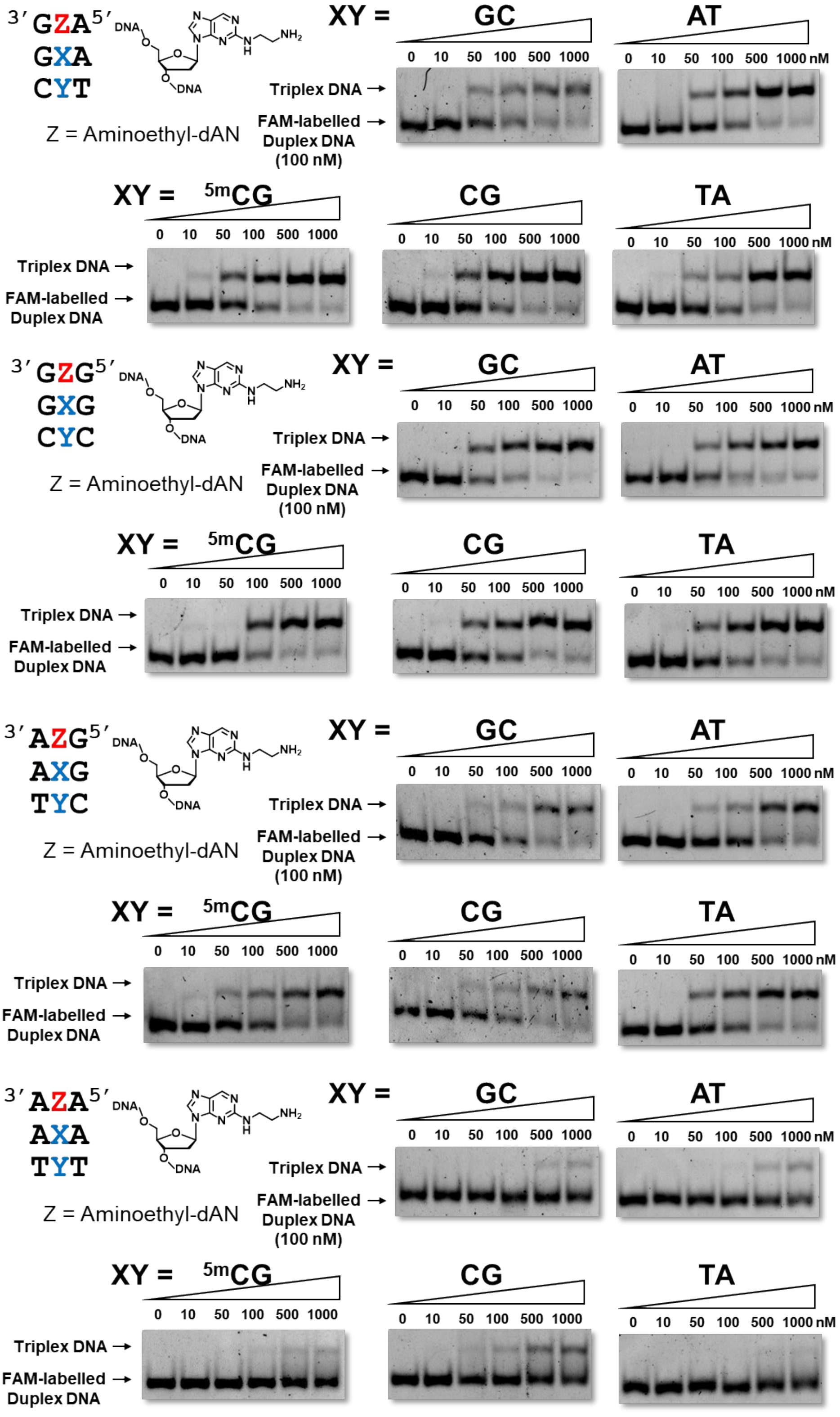

**TFO**	3′-GGAAGG **NZN′** GAGGAGGGA
**Target DNA**	5′-GAGGGAAGG **NXN′** GAGGAGGGAAGC
	3′-CTCCCTTCC **MYM′** CTCCTCCCTTCG-FAM

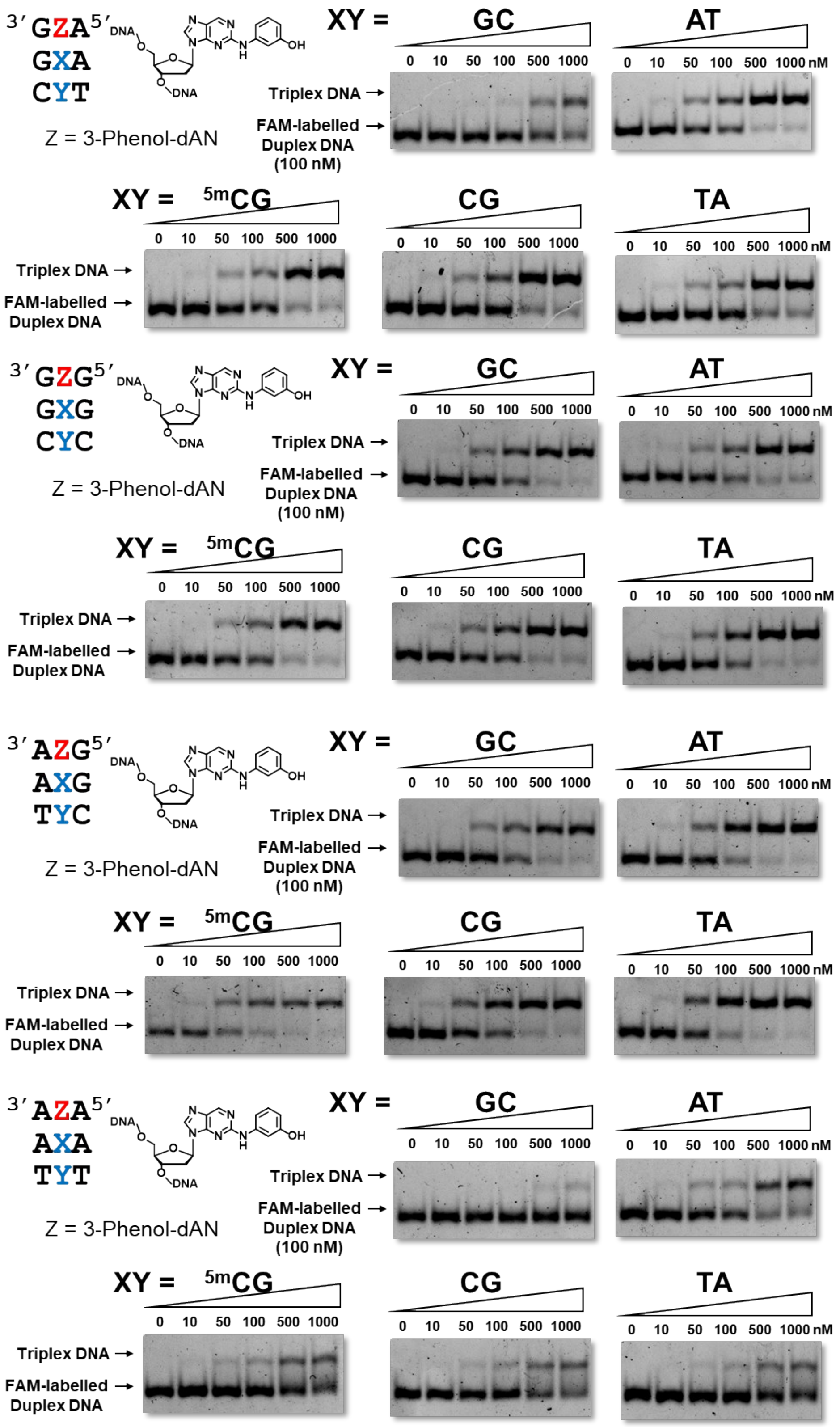

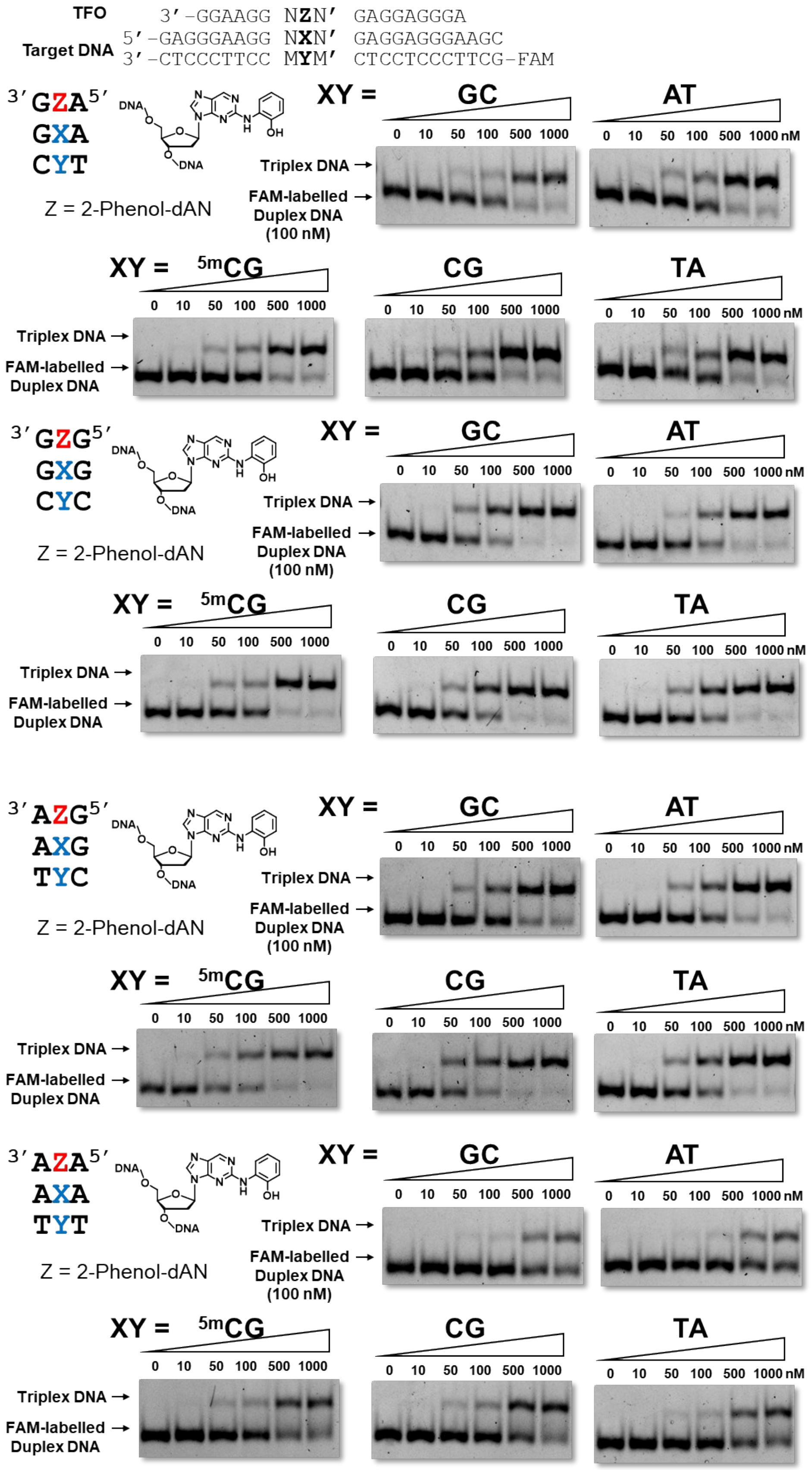
TFO 3′-GGAAGG NZN′ GAGGAGGGA
Target DNA 5′-GAGGGAAGG NXN′ GAGGAGGGAAGC
3′-CTCCCTTCC MYM′ CTCCTCCCTTCG-FAM
3′GZA5′
GXA
CYT
Z = 2-Phenol-dAN
3′GZG5′
GXG
CYC
3′AZG5′
AXG
TYC
3′AZA5′
AXA
TYT
DNA
OH
XY = GC
XY = AT
XY = 5mCG
CG
TA
0 10 50 100 500 1000 nM
Triplex DNA
FAM-labelled Duplex DNA (100 nM)

Figure S5 Gel results of confirmation of triplex formation.

Conditions: The mixture of FAM-labeled Py-strand (24 mer; 100 nM) or Pu-strand (24 mer; 100 nM) or TFO ((A) and (B): 18 mer; 0-1000 nM or (C): 500 nM) was incubated in buffer containing 20 mM Tris-HCl and 20 mM $MgCl_2$ at 37˚C and pH 7.5. Electrophoresis was performed using a 10% non-denatured polyacrylamide gel. (A) Target duplex containing mismatch sites in the natural DNA triplet. (B) Target purine strand oriented parallel to TFO. (C) The mixture of each component one by one. (D) The mixture of each component using reverse-oriented Py-strand and Pu-strand.

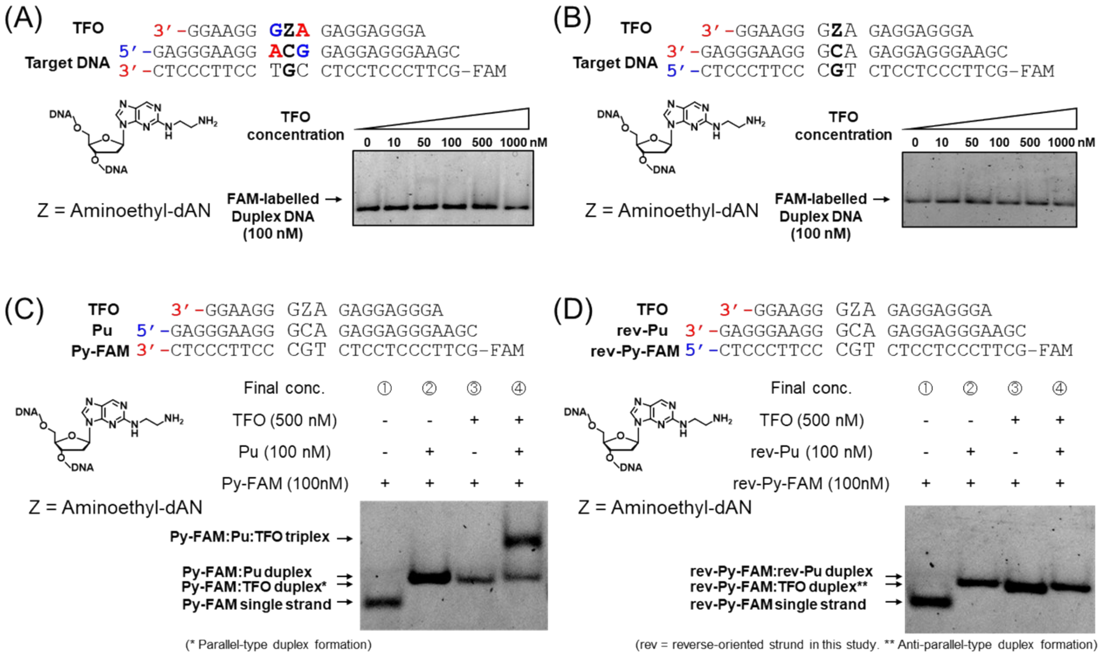

Figure S6 Optimized structures of the dAN-derivatives/^{5m}CG base triplet.

(A) aminoethyl-dAN/^{5m}CG, (B) 3-phenol-dAN/^{5m}CG and (C) 2-phenol-dAN/^{5m}CG, DFT at

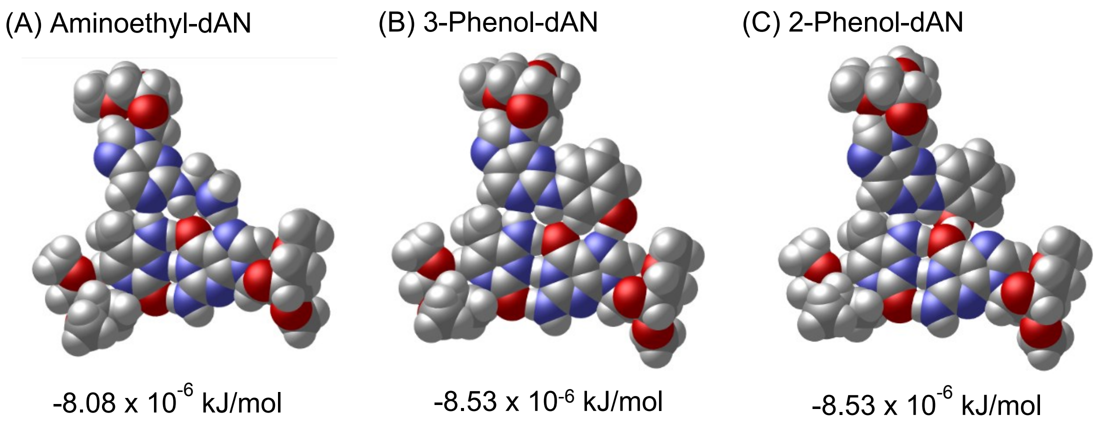

B3LYP/6-31 level.

Figure S7 Molecular modeling of triplex DNA formation.

This triplex DNA includes aminoethyl-dAN/^{5m}CG or CG base triplet under Amber99 force field using HyperChem (7.52). At the end of the calculation, three triplet base pairs are shown as snapshots. The part of the triplet base pair of the artificial nucleoside analogue is shown by the CPK model, and the base triplet structures on both sides are shown in green. (A) Complex containing Aminoethyl-dAN and ^{5m}CG base pair. (B) Complex containing Aminoethyl-dAN and CG base pair.

(A) Aminoethyl-dAN/^{5m}CG (5’-AZG-3’ sequence)

View from the 5‘-side of the TFO

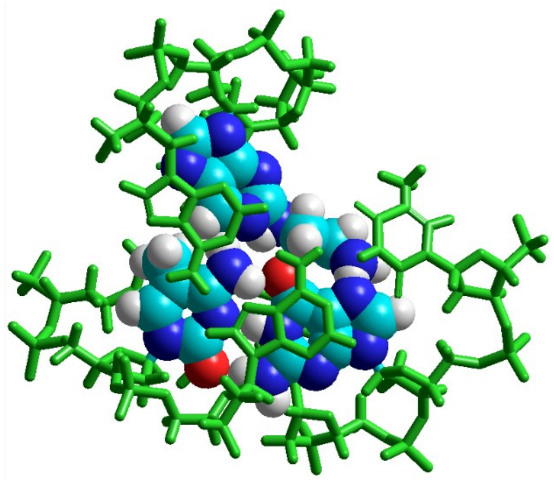

View from the 3‘-side of the TFO

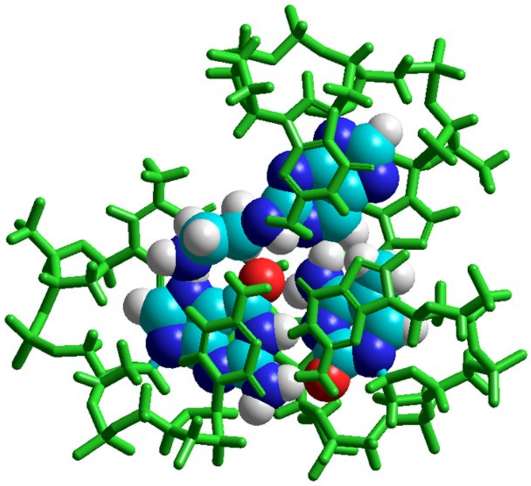

(B) Aminoethyl-dAN/CG (5’-AZG-3’ sequence)

View from the 5‘-side of the TFO

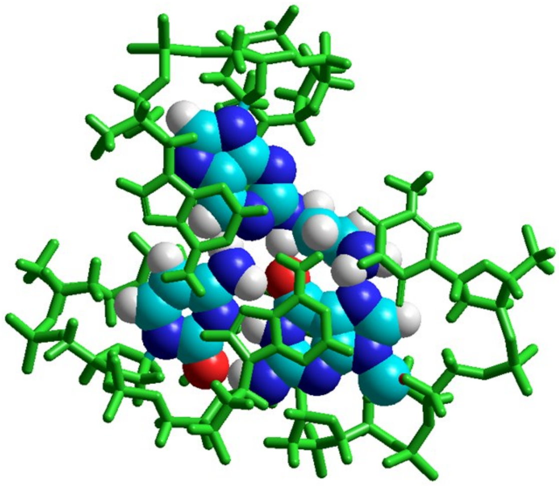

View from the 3‘-side of the TFO

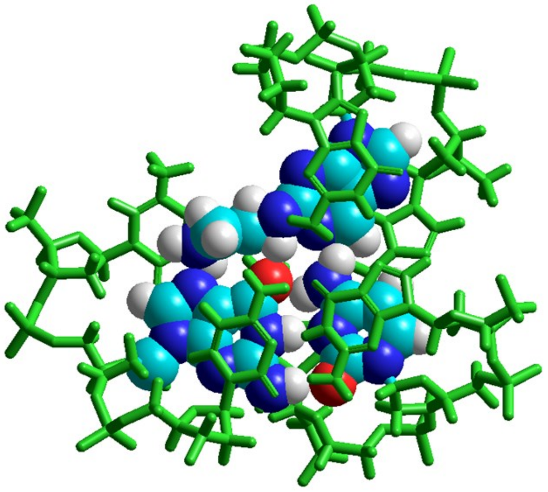

Supplement: RA-011-D1RA02831F-s001 [file RA-011-D1RA02831F-s001.pdf]
